# The CRISPR effector Cam1 mediates membrane depolarization for phage defence

**DOI:** 10.1038/s41586-023-06902-y

**Published:** 2024-01-10

**Authors:** Christian F. Baca, You Yu, Jakob T. Rostøl, Puja Majumder, Dinshaw J. Patel, Luciano A. Marraffini

**Affiliations:** 1 Laboratory of Bacteriology, The Rockefeller University, New York, NY USA; 2 Tri-Institutional PhD Program in Chemical Biology, Weill Cornell Medical College, Rockefeller University and Memorial Sloan Kettering Cancer Center, New York, NY USA; 3 Structural Biology Program, Memorial Sloan Kettering Cancer Center, New York, NY USA; 4 Howard Hughes Medical Institute, The Rockefeller University, New York, NY USA; 5 Present Address: MRC Centre for Molecular Bacteriology and Infection, Imperial College London, London, UK

**Keywords:** Bacterial genetics, X-ray crystallography

## Abstract

Prokaryotic type III CRISPR–Cas systems provide immunity against viruses and plasmids using CRISPR-associated Rossman fold (CARF) protein effectors^1–5^. Recognition of transcripts of these invaders with sequences that are complementary to CRISPR RNA guides leads to the production of cyclic oligoadenylate second messengers, which bind CARF domains and trigger the activity of an effector domain^6,7^. Whereas most effectors degrade host and invader nucleic acids, some are predicted to contain transmembrane helices without an enzymatic function. Whether and how these CARF–transmembrane helix fusion proteins facilitate the type III CRISPR–Cas immune response remains unknown. Here we investigate the role of cyclic oligoadenylate-activated membrane protein 1 (Cam1) during type III CRISPR immunity. Structural and biochemical analyses reveal that the CARF domains of a Cam1 dimer bind cyclic tetra-adenylate second messengers. In vivo, Cam1 localizes to the membrane, is predicted to form a tetrameric transmembrane pore, and provides defence against viral infection through the induction of membrane depolarization and growth arrest. These results reveal that CRISPR immunity does not always operate through the degradation of nucleic acids, but is instead mediated via a wider range of cellular responses.

## Abstract (web summary)

The prokaryotic non-enzymatic effector protein Cam1 mediates CRISPR immunity by binding tetra-adenylate second messengers and forming a pore in the membrane that induces membrane depolarization and growth arrest.

## Main

**Extended Data Fig. 1:**
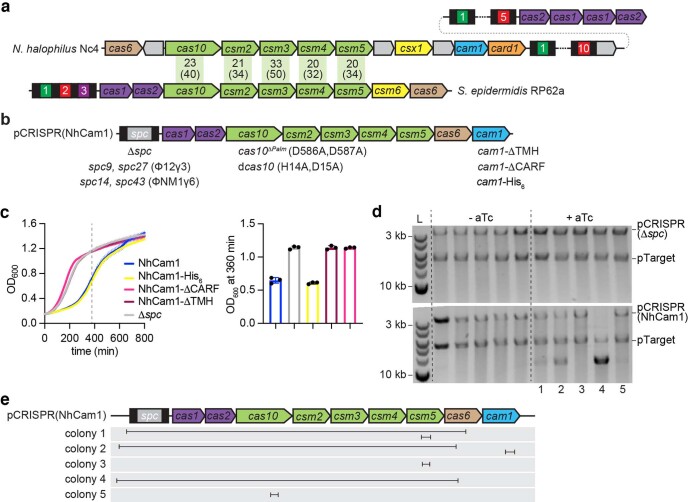
NhCam1 mediates growth arrest in staphylococci. (**a**) Comparison of the type III-A systems of *Nitrosococcus halophilus* Nc4 and *Staphylococcus epidermidis* RP62. Black boxes, CRISPR repeats; colored and numbered boxes, CRISPR spacers. Numbers indicate the % homology at the amino acid sequence level. Numbers in parenthesis indicate the % homology at the DNA sequence level. (**b**) Genetic modifications of the *S. epidermidis* RP62 type III-A CRISPR locus cloned into different pCRISPR plasmids. Insertion of different spacer sequences, amino acid substitutions and domain deletions are indicated. (**c**) Growth of staphylococci carrying pTarget and pCRISPR variants, measured as OD_600_ after the addition of aTc in the absence of antibiotic selection for pTarget. Dotted line marks the OD_600_ value at 395 min, used to generate the bar graph. In both graphs the mean of three biological triplicates, ±s.e.m., is reported. (**d**) Agarose gel electrophoresis of linearized plasmids purified from five different colonies obtained in the final data point of the experiment shown in Fig. 1d. “L”, DNA ladder. Image is representative of two technical replicates. (**e**) Schematic of the DNA sequencing results obtained after NGS of the pCRISPR(NhCam1) plasmids purified from the five colonies obtained in the presence of aTc, shown in panel (**d**). Lines represent the regions of the CRISPR locus not found in these plasmids (presumably deleted). Source Data

CRISPR loci and their associated cas genes encode an RNA-guided mechanism to destroy the nucleic acids of prokaryotic invaders, such as phages^8^ and plasmids^9^. CRISPR loci consist of short (approximately 30–40 bp) DNA repeats separated by equally short spacer sequences that match the genomes of infecting phages and plasmids^10–12^. Transcription and processing of spacers generate the CRISPR RNA (crRNA) guide^13–15^, which associates with Cas nucleases to recognize and cleave complementary targets produced during infection and initiate the CRISPR–Cas immune response^16–18^. Depending on their cas gene content, CRISPR–Cas systems can be classified into six different types^19^. Of these, type III systems display an elaborate targeting mechanism whereby the CRISPR RNA (crRNA)-guided Cas10 complex recognizes an invader’s transcripts to trigger a diverse set of responses. The HD domain of Cas10 degrades single stranded DNA (ssDNA) non-specifically^20^, most probably within or near the transcription bubble generated by the transcription of the target RNA^21^. The Palm domain of Cas10 synthesizes 3′–5′ cyclic oligoadenylate molecules^6,7^ (cA_*n*_, where *n* is the number of adenylates in the ring). These act as second messengers that bind CARF domain proteins encoded by type III loci. CARF domains are fused to an effector domain whose activity is triggered upon cA_*n*_ binding. All CARF effectors described so far function as cA_*n*_-activated nucleases that cleave RNA and/or DNA in a non-sequence-specific manner^1–5^ and mediate growth arrest of the infected cell^22,23^, preventing viral propagation. Previous bioinformatic studies investigating the gene content of type III systems have identified many uncharacterized CARF-containing proteins^24,25^. We studied WP_013033759 (hereafter named Cam1) from the halophilic Gram-negative chromatiaceae *Nitrosococcus halophilus* Nc4 (Extended Data Fig. 1a), which contains a CARF domain fused to a transmembrane domain that does not seem to be capable of degrading nucleic acids, as is the case for all the previously characterized CARF effectors^1–5^.

## Cam1 mediates a growth arrest

**Fig. 1:**
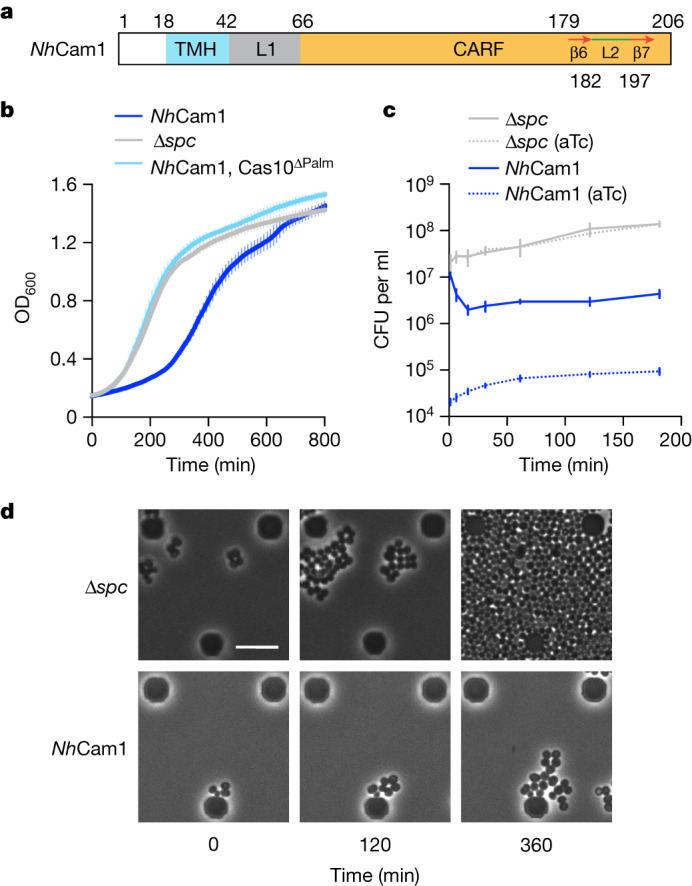
*Nh*Cam1 mediates growth arrest upon activation of type III CRISPR immunity. **a**, Domain architecture of *Nh*Cam1. Numbers indicate amino acids, and β6 and β7 are beta strands. **b**, Growth of staphylococci carrying pTarget and pCRISPR variants, measured as OD_600_ after the addition of aTc in the absence of antibiotic selection for pTarget. Data are mean of three biological triplicates ± s.e.m. **c**, Number of colony-forming units from staphylococcal cultures carrying pCRISPR variants after the addition of aTc. At the indicated times after induction, aliquots were removed and plated on solid medium with or without aTc to count the remaining viable cells. Data are mean of three biological replicates ± s.e.m. **d**, Time-course microscopy of *S. aureus* cells harbouring pTarget and pCRISPR(Δ*spc*) or pCRISPR(*Nh*Cam1) at different times after addition of aTc. Images representative for two biological replicates. Scale bar, 6.8 µm. Source Data

*N. halophilus* Cam1 (hereafter *Nh*Cam1) has a molecular mass of 22.3 kDa and contains 206 amino acids, with an N-terminal transmembrane helix (TMH) predicted to be 24 amino acids in length by deepTMHMM^26^ followed by an equally short linker (L1) and a C-terminal intracellular CARF domain (Fig. 1a). Given the lack of genetic tools for this organism, we decided to study the function of *Nh*Cam1 in the context of the *Staphylococcus epidermidis* RP62 type III-A locus^9^, whose genes display 32–50% sequence similarity to those of the *N. halophilus* Nc4 system (Extended Data Fig. 1a). To this end, we constructed pCRISPR(*Nh*Cam1) by cloning into the staphylococcal plasmid pC194^27^ the *S. epidermidis* RP62 type III-A locus carrying the *cam1* open reading frame but lacking the staphylococcal CARF effector Csm6^28^ (Extended Data Fig. 1b). As controls, we also introduced a pCRISPR plasmid harbouring either the TMH or CARF domains of *Nh*Cam1 alone (*Nh*Cam1-ΔCARF and *Nh*Cam1-ΔTMH, respectively), mutations in the Palm domain of Cas10 that prevent the synthesis of cA_*n*_^6,7^ (D586A–D587A, Cas10^ΔPalm^) and a CRISPR locus without a targeting spacer (Δ*spc*). Each of these plasmids were transformed into *Staphylococcus aureus* RN4220 cells^29^ containing pTarget, a second plasmid producing a target transcript—that is, complementary to the crRNA expressed by the pCRISPR plasmids, under the control of an anhydrotetracycline (aTc)-inducible promoter^23^. In this experimental system, the addition of the inducer triggers the transcription of the target RNA and therefore the production of cA_4_ and cA_6_ by the Palm domain of Cas10. We measured the optical density at 600 nm (OD_600_) of cultures expressing *Nh*Cam1 and a mutant version of Cas10 with an inactive HD domain (H14A/D15A, dCas10) to prevent pTarget degradation^23^. Upon addition of aTc, we detected a marked growth delay that was not observed in cultures lacking an active palm domain (dCas10^ΔPalm^) or in Δ*spc* cultures (Fig. 1b) and required full-length *Nh*Cam1 (Extended Data Fig. 1c and Supplementary Fig. 1; Supplementary Fig. 1 provides unedited source images for all images presented in this study). Similarly to experiments with Csm6^23^ and Card1^3^, we also observed an increase in OD_600_ at around 6 h after addition of aTc, which was a result of the propagation of ‘escaper’ cells carrying non-functional pCRISPR(*Nh*Cam1) plasmids (Extended Data Fig. 1d,e).

The growth impairment mediated by *Nh*Cam1 could be owing to either the arrest or the death of individual cells within the culture. To distinguish between these possibilities, we counted viable staphylococci after addition of aTc, plating aliquots taken from liquid cultures at different times on solid medium lacking the inducer (Fig. 1c). This procedure removes the inducer and allows the formation of colonies from cells that were arrested in the liquid culture, but not from those that died after activation of the type III-A response. *Nh*Cam1 cultures showed an initial decrease in colony counts of one order of magnitude that stabilized after 1 h, demonstrating the presence of a population of viable cells that cannot grow, but do not die, upon activation of cA_*n*_ production. We also counted colonies that were resistant to aTc induction (Fig. 1c) and found that fewer than 10% of the colonies observed on plates lacking aTc come from escaper cells. Using live microscopy, we observed that whereas cells in which the type III-A CRISPR–Cas response is not triggered (Δ*spc*) grow continuously (Fig. 1d and Supplementary Video 1), cells that express *Nh*Cam1 proliferate at a very low rate (Fig. 1d and Supplementary Video 2). Together, these results demonstrate that when type III immunity is activated, *Nh*Cam1 generates either a permanent or temporary growth arrest of the host that does not result in cell lysis.

## *Nh*Cam1 binds cA_4_

**Extended Data Fig. 2:**
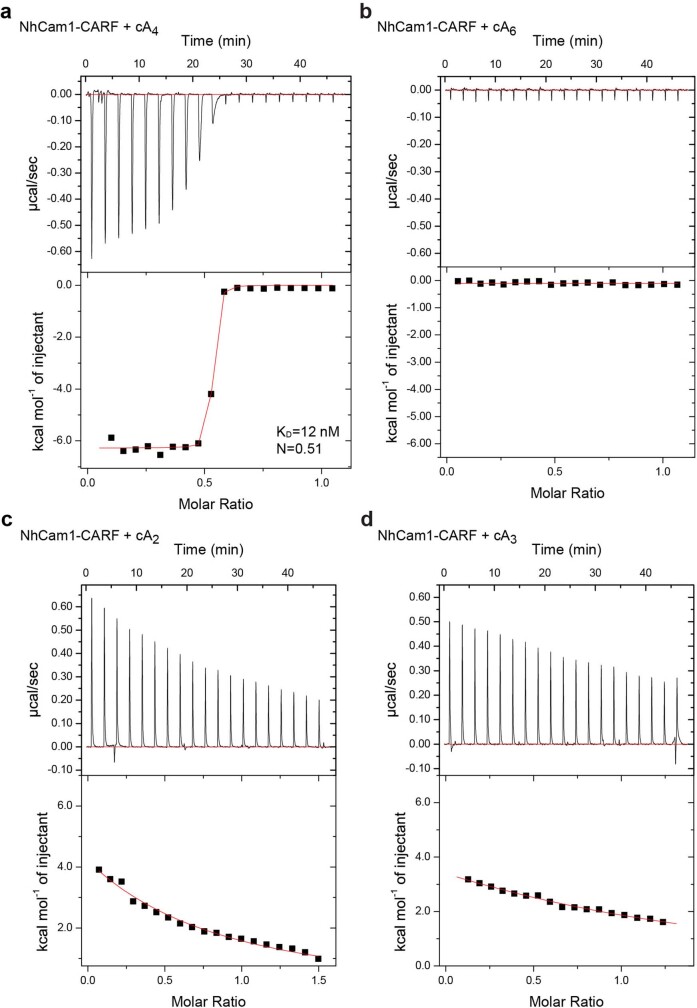
ITC experiments using different cyclic oligoadenylates. (**a**–**d**). ITC plots of the binding of cA_4_ (**a**), cA_6_ (**b**), cA_2_ (**c**) and cA_3_ (**d**) to the CARF (residues 67-206) domain of dimeric NhCam1. Source Data

The above results suggest that type III CRISPR second messengers bind *Nh*Cam1 to trigger growth arrest. To test this, we expressed and purified the *Nh*Cam1 CARF domain (residues 67–206; Fig. 1a) and monitored binding of cA_2_, cA_3_, cA_4_ and cA_6_ oligomers by isothermal calorimetry (ITC). We found that cA_4_ binds to the CARF domain of *Nh*Cam1 with a dissociation constant (*K*_d_) of 12 nM and a stoichiometry of one ligand per CARF domain dimer (*n* = 0.5) (Extended Data Fig. 2a), whereas no binding is observed on addition of cA_6_ (Extended Data Fig. 2b), cA_2_ (Extended Data Fig. 2c) or cA_3_ (Extended Data Fig. 2d).

**Fig. 2:**
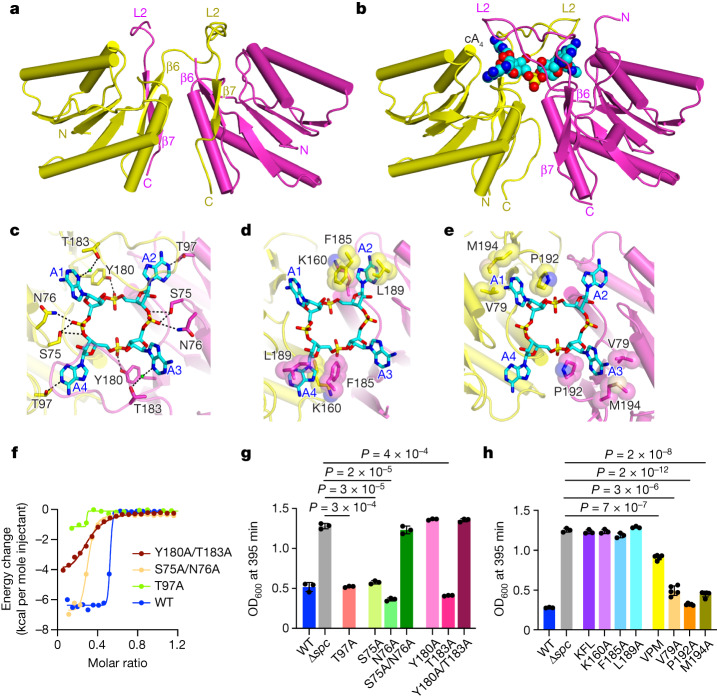
The *Nh*Cam1 CARF domain dimerizes to bind cA_4_. **a**, The 2.2 Å X-ray structure of *Nh*Cam1-CARF, showing the dimeric alignment of the CARF domain of *Nh*Cam1 (residues 42–206) in the apo state. Individual monomers are coloured in yellow and magenta. Note the swapped dimeric topology, in which β6 of one monomer pairs with β7 of the other monomer. **b**, The 2.1 Å X-ray structure of *Nh*Cam1-CARF bound to cA_4_, showing cA_4_ binding within the dimeric alignment of the CARF domain (residues 42–206) of *Nh*Cam1. **c**, Intermolecular hydrogen-bonding alignments between bound cA_4_ and CARF domain residues (residues 42–206) in the X-ray structure of the dimeric cA_4_–*Nh*Cam1 complex. **d**,**e**, Intermolecular hydrophobic contacts involving A2 and A4 (**d**) and A1 and A3 (**e**) with CARF domain residues (residues 42–206) in the X-ray structure of the CARF domain (residues 42–206) of dimeric *Nh*Cam1 in the cA_4_-bound state. **f**, ITC plots comparing the binding affinity of cA_4_ to the CARF domain (residues 67–206) of dimeric *Nh*Cam1 in wild-type (WT) and T97A, S75A/N76A and Y180A/T183A mutant *Nh*Cam1. **g**, Growth of staphylococci carrying pTarget and pCRISPR(*Nh*Cam1) harbouring alanine substitutions of the residues shown in **c**, measured as the OD_600_ value 395 min after addition of aTc. Data are mean of three biological triplicates ± s.e.m. Two-sided *t*-test with Welch’s correction. **h**, As in **g**, but testing alanine substitutions of residues shown in **d** and **e**. KFL, K160A/F185A/L189A. Data are mean of three biological triplicates ± s.e.m. Two-sided *t*-test with Welch’s correction. Source Data

**Extended Data Fig. 3:**
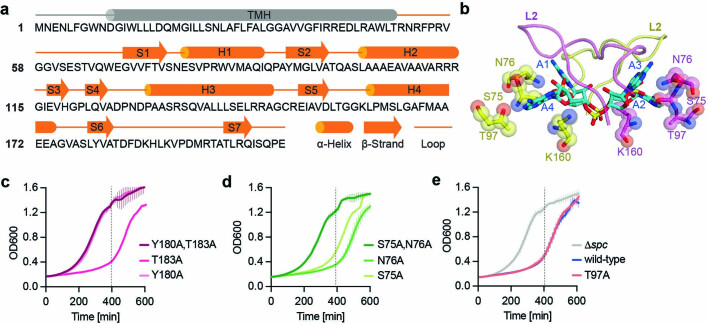
Analysis NhCam1 residues that interact with cA_4_ through hydrogen bonding. (**a**) Sequence alignment of full-length NhCam1, together with secondary structure shown above the sequence. The secondary structure of the TMH helix is predicted while the secondary structure of the CARF domain is based on the x-ray structures of dimeric NhCam1 in the apo- and cA_4_-bound states. (**b**) Positioning of bound cA_4_ on a platform formed by the side chains of Lys160, Thr97, Ser75 and Asn76 in the x-ray structure of the CARF(42-206) domain of dimeric NhCam1 in the cA_4_-bound state. (**c**–**e**) Growth of staphylococci carrying pTarget and pCRISPR(NhCam1) harboring alanine substitutions of the NhCam1 residues shown in Fig. 2c, measured as the OD_600_ value over time. Dotted line marks the OD_600_ value at 395 min, used to generate Fig. 2g. Mean of three biological triplicates, ±s.e.m., is reported. Source Data

We next grew crystals and solved the structure *Nh*Cam1-CARF (residues 42–206) in the absence and presence of bound cA_4_. The 2.2 Å-resolution X-ray structure without the ligand showed that *Nh*Cam1-CARF adopts a domain-swapped dimeric topology, in which the β7 strand of one monomer completes the folding topology of a second monomer by pairing with its β6 strand (Fig. 2a and Extended Data Fig. 3a). Loop L2 connects β6 and β7 in one monomer, but this loop is disordered in the other monomer; loop L1 is disordered in both monomers of the CARF dimer. By contrast, the 2.1 Å X-ray structure of *Nh*Cam1-CARF bound to cA_4_ shows that it adopts a dimeric fold with no domain swap between the monomers (Fig. 2b). Loop L2 adopts different conformations in each monomer of the *Nh*Cam1-CARF dimer in the complex, with the N and C termini being in close proximity in one monomer but far apart in the other monomer, owing to crystal packing forces. The bound cA_4_ is positioned over a platform generated from both monomers, and is further capped by a pair of L2 loops, thereby encapsulating the ligand (Extended Data Fig. 3b and Supplementary Video 3). The bound cA_4_ complex is stabilized by hydrogen-bond interactions between N1 nitrogens of A2 and A4 and the Thr97 side chains of the CARF domain, hydrogen-bond interactions involving the backbone phosphates of bound cA_4_ (Fig. 2c), as well as hydrophobic contacts between the adenosine rings and non-polar side chains of the CARF domain (Fig. 2d,e). The overall topology (Supplementary Fig. 2a), hydrogen-bonding (Supplementary Fig. 2b) and hydrophobic interactions (Supplementary Fig. 2c) involving second messenger binding in *Nh*Cam1 show similarities with cA_4_-bound TsCard1^3^, but not with TsCsm6^5^ or SiCsx1^4^ (Supplementary Fig. 3). The conservation of important residues for these interactions is also low (data not shown).

**Extended Data Fig. 4:**
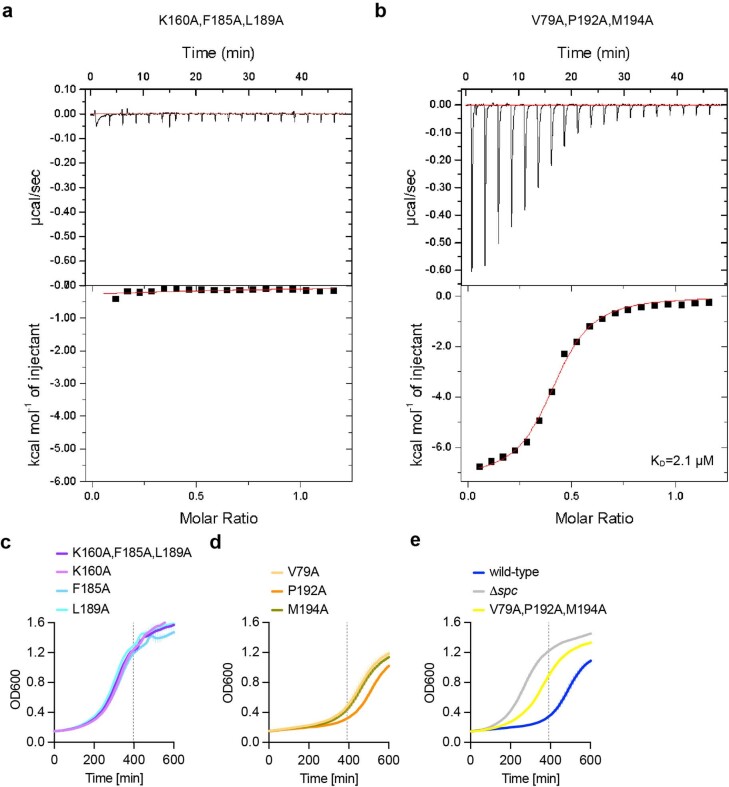
Analysis NhCam1 non-polar residues that interact with cA_4_. (**a**,**b**) ITC plots of the binding of cA_4_ to CARF(67-206) domain of dimeric NhCam1 with K160A + F185A + L189A mutants (**a**) and V79A + P192A + M194A mutants (**b**). (**c**) Growth of staphylococci carrying pTarget and pCRISPR(NhCam1) harboring alanine substitutions of the NhCam1 residues shown in Fig. 2d, measured as the OD_600_ value over time. Dotted line marks the OD_600_ value at 395 min, used to generate Fig. 2h. Mean of three biological triplicates, ±s.e.m., is reported. (**d**–**e**) Same as (**c**) but for the NhCam1 residues shown in Fig. 2e. Mean of three biological triplicates, ±s.e.m., is reported. Source Data

Alanine substitutions of amino acids involved in hydrogen bonds with cA_4_—T97A, S75A/N76A and Y180A/T183A—reduced ligand affinity from 17 nM to 3.74 µM, 4.0 µM and 4.5 µM, respectively (Fig. 2f). In vivo, we found that the Y180A mutation, as well as the S75A/N76A double mutant impaired the ability of *Nh*Cam1 to mediate a growth arrest (Fig. 2g and Extended Data Fig. 3c,d). By contrast, the T97A mutation that severely decreased ligand affinity in vitro (Fig. 2f) produced a delayed growth similar to that induced by wild-type *Nh*Cam1 in vivo (Fig. 2g and Extended Data Fig. 3e). This result suggests that the presence of the TMH domain can alter the cA_4_-binding properties of the CARF domain, most probably in the context of the activation of *Nh*Cam1 function. Triple alanine substitutions of amino acids involved in hydrophobic contacts with the ligand—K160A/F185A/L189A and V79A/P192A/M194A—resulted in a complete absence of ligand binding (Extended Data Fig. 4a), and a reduction of binding affinity to 2.1 µM (Extended Data Fig. 4b), respectively. Consistent with these results, the K160A/F185A/L189A mutant did not induce a growth arrest in vivo (Fig. 2h and Extended Data Fig. 4c), whereas the V79A/P192A/M194A mutant displayed a partial growth phenotype (Fig. 2h and Extended Data Fig. 4d,e). Analysis of single alanine mutants showed that K160, F185 and L189 are all essential for *Nh*Cam1 function in vivo, and are therefore presumably important for cA_4_ binding during the type III-A immune response (Fig. 2h and Extended Data Fig. 4c). By contrast, the single alanine substitutions of V79, P192 or M194 did not affect *Nh*Cam1 activity (Fig. 2h and Extended Data Fig. 4d,e).

**Extended Data Fig. 5:**
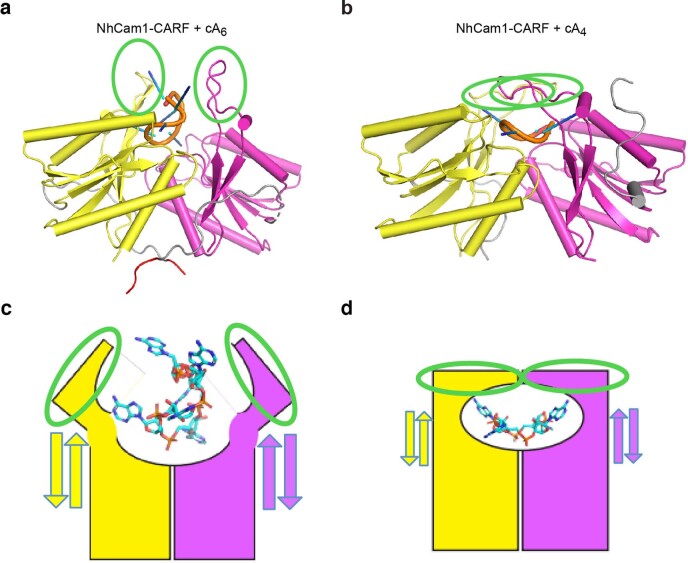
Crystal structure of NhCam1-CARF bound to cA_6_. (**a**,**b**) The 1.9 Å x-ray structure of cA_6_ bound to the dimeric CARF (residues 42-206) domain of NhCam1 (**a**) and the 2.1 Å x-ray structure of cA_4_ bound to the dimeric CARF (residues 42-206) domain of NhCam1 (**b**). Green ovals highlight the loop L2 in the two structures (**c**, **d**) A schematic representation of cA_6_ (**c**) and cA_4_ (**d**) bound to the dimeric CARF domain of NhCam1. Although cA_6_ occupies the same pocket of the NhCam1-CARF dimer that binds cA_4_, only three contiguous adenosines could be fit into the electron density for bound cA_6_ within the binding pocket, while the other three adenosines had poor density and were positioned outside of the pocket, thereby preventing the capping of the bound ligand by the pair of L2 loops.

Finally, despite not observing binding of cA_6_ in our ITC experiments (Extended Data Fig. 2b), we were able to crystallize and solve the 1.9 Å X-ray structure of *Nh*Cam1-CARF (residues 42–206) bound to this ligand. We found that cA_6_ occupies, but does not entirely fit, the same pocket of the *Nh*Cam1-CARF dimer that binds cA_4_ (Extended Data Fig. 5). Together, these results demonstrate that the synthesis of cA_4_ by the Cas10 complex after recognition of a target transcript provides the activating ligand for *Nh*Cam1.

## *Nh*Cam1 is predicted to form a pore

**Fig. 3:**
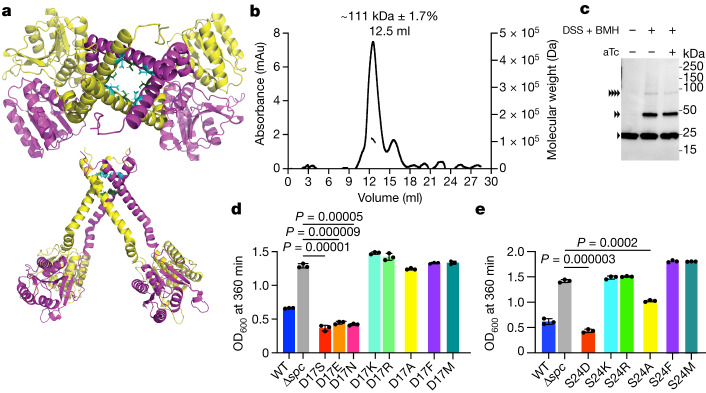
*Nh*Cam1 is predicted to form a tetrameric pore. **a**, Structure of a tetrameric *Nh*Cam1 obtained using AlphaFold2 simulation of different multimeric forms. Two *Nh*Cam1 subunits (yellow and magenta) form a dimer; two of these dimers form a tetrameric pore. Aspartate and serine residues lining the opening of the pore are shown in cyan and green, respectively. **b**, SEC–MALS measurement of the molecular weight of *Nh*Cam1 solubilized into DDM micelles. The measured molecular weight of 111 kDa is close to the calculated molecular weight of tetrameric Cam1 (99.6 kDa). The experiment was repeated for two biological replicates. **c**, Western blot of extracts from staphylococci expressing *Nh*Cam1–His that were treated with DSS and BMH crosslinkers or mock-treated, in the presence or absence of aTc. A primary anti-His_6_ antibody was used on these samples. Arrowheads each represent a single *Mv*Cam1 subunit. Images are representative of three biological triplicates. **d**, Growth of staphylococci carrying pTarget and pCRISPR(*Nh*Cam1) harbouring different D17 substitutions in *Nh*Cam1, measured as the OD_600_ value 395 min after addition of aTc. Data are mean of three biological triplicates ± s.e.m. Two-sided *t*-test with Welch’s correction. **e**, As in **d**, but testing substitutions of S24. Data are mean of three biological triplicates ± s.e.m. Two-sided *t*-test with Welch’s correction. Source Data

**Extended Data Fig. 6:**
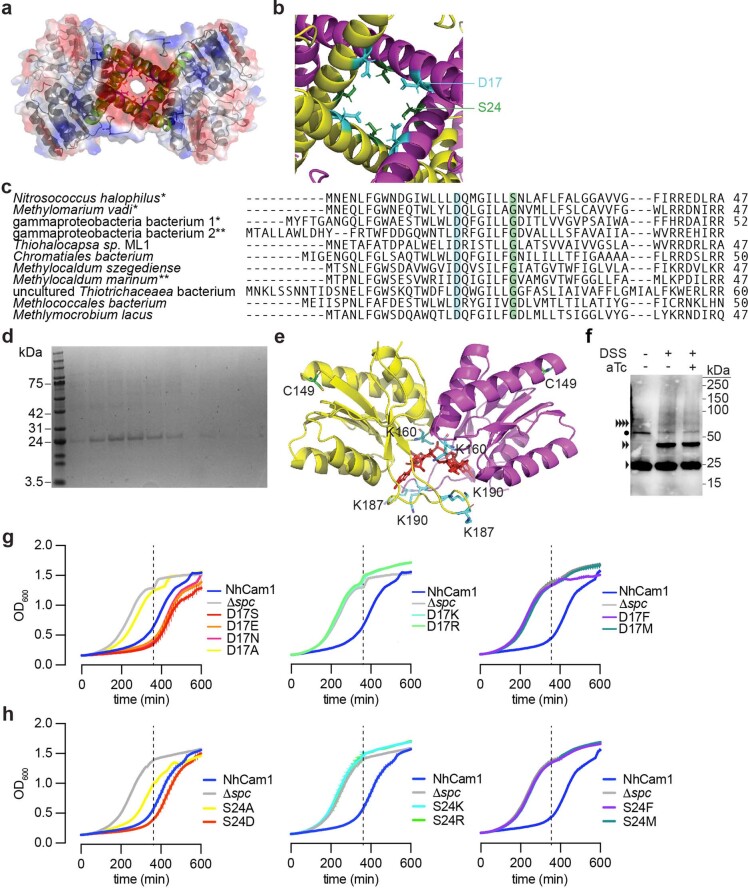
Alphafold model of tetrameric NhCam1. (**a**) Alphafold model of tetrameric NhCam1 structure, showing surface electrostatics in blue (positive charge) and red (negative charge). (**b**) Zoom in of the predicted pore formed by the NhCam1 tetramer, showing the D17 and S24 pore-lining residues in cyan and green, respectively. (**c**) Sequence alignment of the N-terminal, transmembrane helix, domains of different Cam1 homologs, showing conservation for the D17 (cyan), but not for the S24 (green), pore-lining residues. (*) indicates Cam1 homologs that showed activity in our experimental set up; (**) indicates those that were tested but showed no activity. (**d**) SDS-PAGE of NhCam1 purified and solubilized into β-DDM micelles, used for the SEC-MALS experiment of Fig. 3b. Image is representative of two biological replicates. (**e**) Structure of the NhCam1-CARF dimer showing the lysine and cysteine residues available for crosslinking. (**f**) Western blot of extracts from staphylococci expressing NhCam1-His that were treated or (mock-treated) with the DSS crosslinker, in the presence or absence of aTc. A primary anti-hexahistidine antibody was used to detect NhCam1-His. Image is representative of three biological triplicates. (**g**) Growth of staphylococci carrying pTarget and pCRISPR(NhCam1) harboring different substitutions of the D17 residue, measured as the OD_600_ value over time. Dotted line marks the OD_600_ value at 395 min, used to generate Fig. 2g. Mean of three biological triplicates, ±s.e.m., is reported. (**h**) Growth of staphylococci carrying pTarget and pCRISPR(NhCam1) harboring different substitutions of the S24 residue, measured as the OD_600_ value over time. Dotted line marks the OD_600_ value at 395 min, used to generate Fig. 2h. Mean of three biological triplicates, ±s.e.m., is reported. Source Data

Owing to the difficulties of obtaining a structure of full-length *Nh*Cam1, we performed AlphaFold2 simulations^30^ of different multimeric forms. One of the structural predictions suggested the formation of a tetrameric complex comprising two separate CARF dimers and four N-terminal transmembrane α-helices organized into a pore lined with residues D17 (which confers a negative charge to the pore) and S24 (Fig. 3a and Extended Data Fig. 6a,b). We identified 56 different Cam1 homologues (Extended Data Table 1), which we used to perform a MUSCLE protein sequence alignment^31^, finding that D17, but not S24, is highly conserved (Extended Data Fig. 6c). Notably, the apo *Nh*Cam1-CARF dimer crystal structure aligned to the CARF domain of the model with a root mean-squared deviation of 0.44 Å.

To evaluate the formation of multimeric structures, we expressed full-length *Nh*Cam1 with the addition of a hexahistidine tag and a TEV protease sequence (MGSS–His_6_–TEV–*Nh*Cam1, 24.9 kDa). We then solubilized and purified the protein using *n*-dodecyl-β-d-maltoside (DDM) micelles (Extended Data Fig. 6d). Next, we analysed this preparation using size-exclusion chromatography–multi-angle light scattering^32^ (SEC–MALS) and observed a major elution peak corresponding to a molecular weight of 111 kDa ± 1.7% (Fig. 3b), a value consistent with the formation of a tetramer in vitro. Next, we used chemical crosslinking to determine the oligomeric state of *Nh*Cam1 in vivo. We used a mix of the membrane-permeable, amine-reactive disuccinimidyl suberate^33^ (DSS) and the membrane-permeable, sulfhydryl-reactive bismaleimidohexane^34^ (BMH) for simultaneous crosslinking of lysine and cysteine side chains, respectively, in *Nh*Cam1 (Extended Data Fig. 6e). Western blot of lysates of staphylococci expressing *Nh*Cam1–His revealed species with higher molecular weight in the presence of the crosslinker (Fig. 3c) corresponding to a dimer and a tetramer. We corroborated this result by performing the crosslinking with DSS alone (Extended Data Fig. 6f). Finally, we found that production of cA_4_ in staphylococci did not alter any of the crosslinking patterns (Fig. 3c and Extended Data Fig. 6f), a result that suggest that ligand binding activates a pre-assembled pore, rather than triggering the assembly of Cam1 monomers.

Mutagenesis analysis of the putative pore-lining residues showed that only mutations that preserve a negatively charged or polar side chain position 17 (serine and asparagine) maintain *Nh*Cam1 function in growth arrest (Fig. 3d and Extended Data Fig. 6g). By contrast, substitution of D17 with basic (lysine or arginine) or large hydrophobic (phenylalanine or methionine) residues, or alanine disrupted *Nh*Cam1 activity. Similarly, although the S24D substitution maintained *Nh*Cam1 function, changes of this residue to lysine, arginine, phenylalanine or methionine eliminated the growth arrest phenotype, or partially abrogated it in the case of the S24A mutant (Fig. 3e and Extended Data Fig. 6h). Altogether, these experimental data support a structural model of a tetrameric pore lined with negatively charged or polar residues for *Nh*Cam1.

## *Nh*Cam1 causes membrane depolarization

**Fig. 4:**
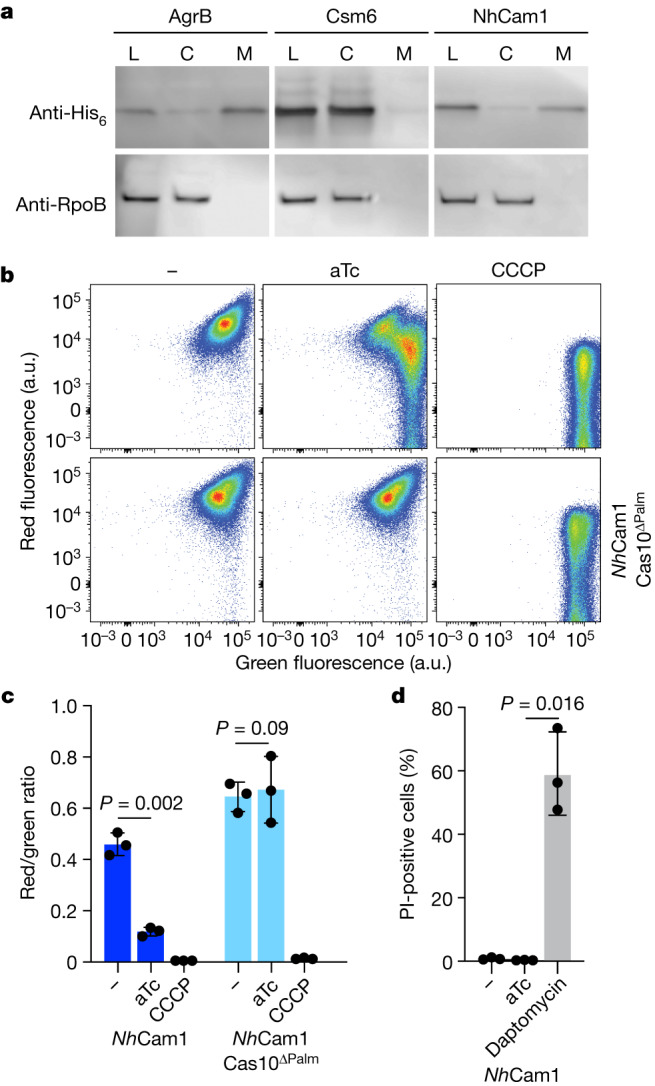
*Nh*Cam1 mediates membrane depolarization. **a**, Western blot of cellular fractions. Staphylococci harbouring plasmids encoding C-terminally hexahistidine-tagged AgrB, Csm6 or *Nh*Cam1 were grown to an OD_600_ of 0.5. Cells were disrupted to collect the total lysate fraction (L), which was subsequently fractionated by ultracentrifugation to obtain cytosolic (C) and membrane (M) fractions. Samples were blotted with a primary anti-His_6_ antibody. Anti-RpoB was used as a loading control. The protein molecular weight ladder is shown in Supplementary Fig. 1b. The experiment was repeated for two biological replicates. **b**, Flow cytometry of *S. aureus* cells harbouring various pCRISPR constructs and stained with DiOC_2_(3), collected 30 min after addition of aTc or the depolarizing agent CCCP. a.u., arbitrary units; green fluorescence, emission of 515 nm upon 488 nm excitation; red fluorescence, emission of 610 nm upon 561 nm excitation. Each plot is representative of approximately 100,000 cells. Blue points represent low density of events while red points represent higher density of events. **c**, Quantification of flow cytometry in **b**. Two-sided *t*-test with Welch’s correction. The ratio of red to green fluorescence was calculated from the mean fluorescence intensities of red and green channels. Data are mean of three biological triplicates ± s.e.m. Two-sided Welch’s *t*-test. **d**, Quantification of propidium iodide (PI) fluorescence measured after addition of either aTc or daptomycin to staphylococci harbouring pTarget and pCRISPR(*Nh*Cam1). Data are mean of three biological triplicates ± s.e.m. Two-sided Welch’s *t*-test. At least 150 cells were analysed for each biological replicate to calculate the percentage of propidium iodide-positive cells. Source Data

The prediction that *Nh*Cam1 TMH domains form a tetrameric pore suggests a membrane association. We therefore performed western blot analysis to determine the subcellular localization of *Nh*Cam1. We tagged *Nh*Cam1—as well as Csm6 and AgrB, which localize to the cytoplasm and membrane fractions, respectively^28,35^—with a C-terminal hexahistidine tag (Extended Data Fig. 1a). *Nh*Cam1–His and AgrB–His, but not Csm6–His, were present in the membrane fraction (Fig. 4a), demonstrating that *Nh*Cam1 is a membrane-bound protein in staphylococci.

**Extended Data Fig. 7:**
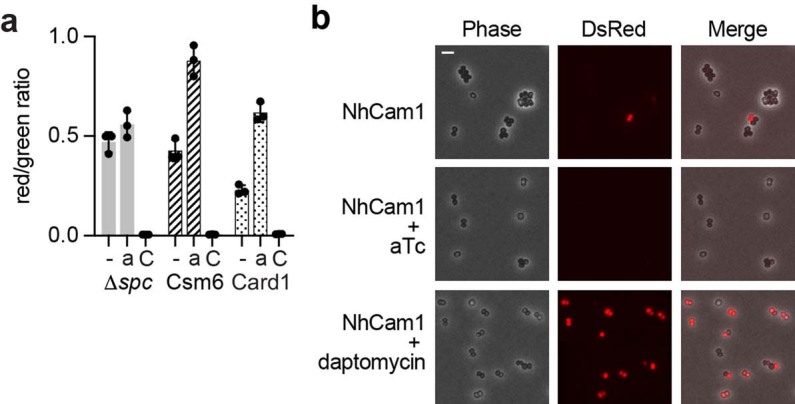
Analysis of NhCam1 function in membrane depolarization. (**a**) Quantification of flow cytometry of *S. aureus* cells harboring pCRISPR constructs without a targeting spacer but expressing NhCam1 (Δ*spc*) or with a spacer targeting a transcript produced by pTarget but expressing the nucleases Csm6 or Card1. Cells were stained with DiOC_2_(3) and collected 30 min after addition of either aTc or the depolarizing agent CCCP. The ratio of red to green fluorescence was calculated from the mean fluorescence intensities (MFIs) of red and green channels. “a”, aTc; “C”, CCCP. Mean of three biological triplicates, ±s.e.m. is reported. (**b**) Fluorescent microscopy of staphylococci harboring pTarget and pCRISPR(NhCam1) in the presence of propidium iodine, either untreated or after the addition of aTc or daptomycin. Images are representative of three biological triplicates. Scale bar is 3.0 μM. Source Data

Considering its localization and oligomerization, we hypothesized that *Nh*Cam1 might cause cell toxicity via membrane depolarization, a mechanism previously associated with phage lysis and CRISPR defence, mediated by pinholins^36^ and Csx28^37^, respectively. To test this, we stained cells undergoing *Nh*Cam1-mediated growth arrest with the membrane potential indicator dye 3,3′-diethyloxacarbocyanine iodide (DiOC_2_(3)). DiOC_2_(3) primarily emits green fluorescence and shifts to red in the presence of proper polarization^38^. As a positive control, we used the membrane potential disruptor carbonyl cyanide *m*-chlorophenylhydrazone^38^ (CCCP). Using flow cytometry, we found that cA_4_ production decreased the shift to red fluorescence only in the presence of an intact Palm domain in Cas10 (Fig. 4b,c), although leaky target expression in the absence of aTc generated a lower red/green ratio in cells expressing wild-type Cas10. Notably, this reduction was not observed in cells expressing different cA_4_-binding CARF effectors, such as Csm6^23^ and Card1^3^, or in cells lacking a targeting spacer (Δ*spc*) (Extended Data Fig. 7a). Finally, we tested whether this depolarization is a consequence of a general, non-specific membrane disruption by comparing the effects of *Nh*Cam1 activation with those of daptomycin, an antibiotic that inserts into the bacterial membrane, altering its biophysical properties and inducing ion leakage^39,40^. We performed fluorescence microscopy in the presence of propidium iodide, which emits red fluorescence upon intercalation with DNA when the cell membrane is sufficiently disrupted to allow dye intake, but should not be internalized by the formation of a small pore^37^. In contrast to daptomycin, *Nh*Cam1 activation upon addition of aTc to the cultures did not lead to an increase in fluorescent staphylococci (Fig. 4d and Extended Data Fig. 7b). These results are also aligned with the findings presented in Fig. 1 showing that *Nh*Cam1 arrests the growth of, but does not lyse, host cells. Together, these observations suggest that the toxicity imposed by *Nh*Cam1 activation is probably due to its ability to depolarize the bacterial membrane without compromising its integrity.

## Cam1 homologues mediate growth arrest

**Extended Data Fig. 8:**
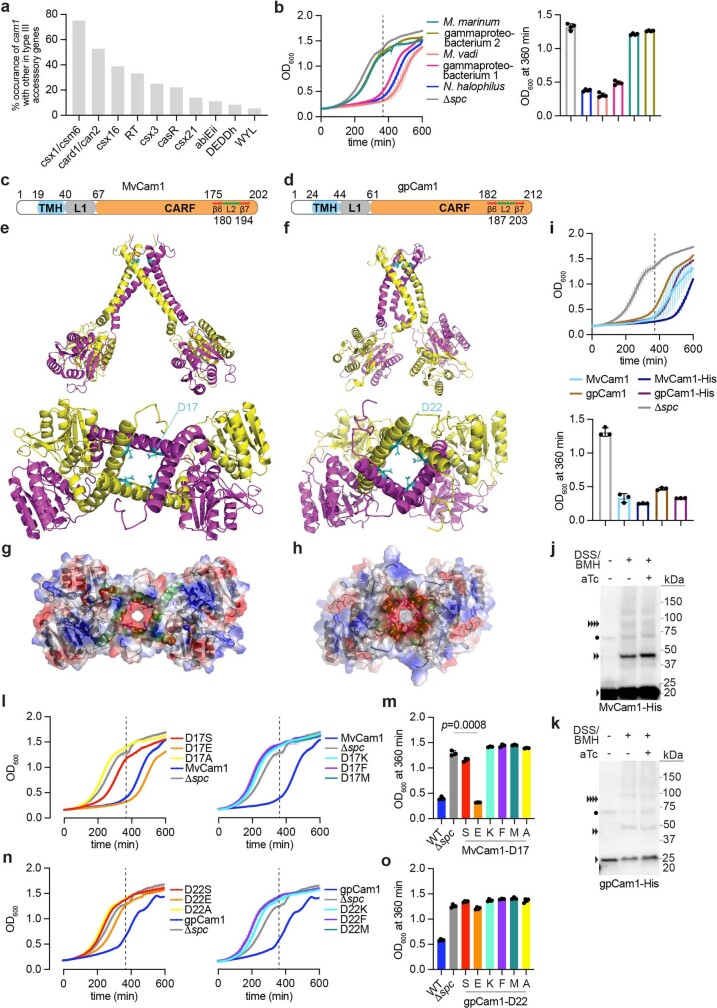
Cam1 homologs mediate growth arrest. (**a**) Analysis of co-occurrence of Cam1 with other known type III accessory effectors, reported as the % of genomes containing Cam1 that also contain any of the effectors shown in the *x* axis. (**b**) Growth of staphylococci carrying pTarget and pCRISPR plasmids harboring different Cam1 homologs, measured as OD_600_ after the addition of aTc in the absence of antibiotic selection for pTarget. Dotted line marks the OD_600_ value at 395 min, used to generate the bar graph. In both graphs the mean of three biological triplicates, ±s.e.m., is reported. (**c**) Domain architecture of *Methylomarinum vadi* Cam1, MvCam1. Numbers indicate amino acids; TMH, transmembrane helix; L1, L2, linkers; CARF, CRISPR-associated Rossman fold domain; β6, β7, beta strands. (**d**) Same as (**c**) but for gammaproteobacterium bacterium Cam1, gpCam1. (**e**) Structure of a tetrameric MvCam1 obtained using Alphafold2 simulation of different multimeric forms. Two MvCam1 subunits (yellow and magenta) form a dimer; two of these dimers form a tetrameric pore. Aspartate residues (D17) lining the opening of the pore are shown in cyan. (**f**) Same as (**e**) but for gpCam1, showing the location of D22 within the pore. (**g**) Alphafold model of tetrameric MvCam1 structure, showing surface electrostatics in blue (positive charge) and red (negative charge). (**h**) Same as (**g**) but for gpCam1. (**i**) Same as (**b**) but using staphylococci that carry pCRISPR plasmids expressing MvCam1, gpCam1 or their His6-tagged versions. (**j**) Western blot of extracts from staphylococci expressing MvCam1-His that were treated or (mock-treated) with DSS and BMH crosslinkers, in the presence or absence of aTc. A primary anti-hexahistidine antibody was used on these samples. Arrowheads represent a single MvCam1 subunit. The circle indicates a non-specific band. Image is representative of two biological replicates. (**k**) Same as (**j**) but using extracts of staphylococci expressing gpCam1-His. (**l**) Growth of staphylococci carrying pTarget and pCRISPR(MvCam1) harboring different substitutions of the D17 residue, measured as the OD_600_ value over time. Dotted line marks the OD_600_ value at 395 min. Mean of three biological triplicates, ±s.e.m., is reported. (**m**) OD_600_ value after 395 min of addition of aTc obtained in (**l**). Mean of three biological triplicates, ±s.e.m., is reported. *p* values, obtained with a two-sided *t*-test with Welch’s correction, are shown. (**n**) Growth of staphylococci carrying pTarget and pCRISPR(gpCam1) harboring different substitutions of the D22 residue, measured as the OD_600_ value over time. Dotted line marks the OD_600_ value at 395 min. Mean of three biological triplicates, ±s.e.m., is reported. (**o**) OD_600_ value after 395 min of addition of aTc obtained in (**n**). Mean of three biological triplicates, ±s.e.m., is reported. Source Data

Analysis of the 56 Cam1 homologues showed that, as expected, most are present within type III CRISPR–Cas loci and are associated with other predicted CARF-containing effectors (Extended Data Table 1 and Extended Data Fig. 8a). We tested five of these homologues for activity in our *S. aureus* experimental system and found two, from *Methylomarium vadi* (*Mv*Cam1) and an unknown Gammaproteobacterium (gpCam1), that mediated growth arrest (Extended Data Fig. 8b). These homologues exhibit less than 50% sequence identity to *Nh*Cam1, approximately 47% for *Mv*Cam1 and 42% for gpCam1, and to each other (40% identity). In spite of their low sequence identity, both homologues share the domain architecture of *Nh*Cam1 (Extended Data Fig. 5c,d) and a predicted a tetrameric pore-forming structure (Extended Data Fig. 5e,f) with a negatively charged pore (Extended Data Fig. 8g,h) lined by a conserved aspartic acid residue (D17 for *Mv*Cam1 and D22 for gpCam1; Extended Data Figs. 6c and 8e,f). We validated these predictions using crosslinking and western blot of C-terminal hexahistidine-tagged versions of these homologues that were able to mediate growth arrest (Extended Data Fig. 8i). Similarly to *Nh*Cam1–His, treatment of cells with DSS and BMH led to the detection of species with molecular weights that corresponded to dimeric and tetrameric forms of the Cam1 homologues, a pattern that was not affected by aTc (Extended Data Fig. 8j,k).

Finally, as observed for *Nh*Cam1, mutations of the conserved aspartates to residues that invert the charge (lysine), insert a bulky residue (phenylalanine or methionine), or to alanine, abrogated the ability of Cam1 homologues to mediate growth arrest (Extended Data Fig. 8l,o). In the case of the substitutions for amino acids with negative side chains, only the D17E mutation, but not D17S, was tolerated in *Mv*Cam1 (Extended Data Fig. 8l,m), and neither D22E nor D22S variants of gpCam1 mediated growth arrest during the type III-A response (Extended Data Fig. 8n,o). Provided that the pore models are correct, the lack of disruption of *Mv*Cam1 and gpCam1 function could be owing to the differences in diameter of the putative pores: 9.0 Å for *Nh*Cam1, 7.3 Å for *Mv*Cam1 and 7.5 Å for gpCam1. It is conceivable that the function of smaller pores is more sensitive to changes in the size of substituted amino acid side chains. These results together with the western blot data support the AlphaFold model of a tetrameric pore for Cam1 homologues.

## Cam1 prevents phage replication

**Fig. 5:**
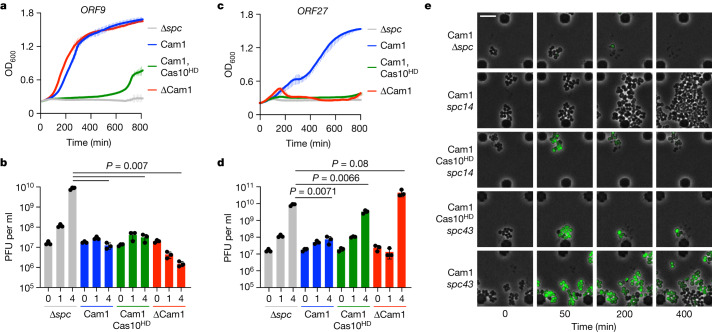
*Nh*Cam1 mediates immunity against phage infection during the type III CRISPR response. **a**, Growth of staphylococci carrying different pCRISPR constructs targeting the *ORF9* transcript of Φ12γ3, measured as OD_600_ after infection at an MOI of approximately 5. Data are mean of three biological triplicates ± s.e.m. Cas10^HD^, Cas10 carrying inactivating mutations in the HD domain. **b**, Number of plaque-forming units in staphylococcal cultures harbouring different pCRISPR constructs programmed to target the *ORF9* transcript, at the indicated times after infection with Φ12γ3 at an MOI of approximately 1. Data are mean of three biological replicates ± s.e.m. Two-sided *t*-test with Welch’s correction. **c**, As in **a**, but with pCRISPR constructs targeting the *ORF27* transcript. **d**, As in **b**, but with pCRISPR constructs targeting the *ORF27* transcript. Data are mean of three biological triplicates ± s.e.m. Two-sided *t*-test with Welch’s correction. **e**, Time-course microscopy of *S. aureus* harbouring different pCRISPR constructs after infection with ΦNM1γ6-GFP. Images are representative of two biological replicates. Scale bar, 3.0 μm. Source Data

**Extended Data Fig. 9:**
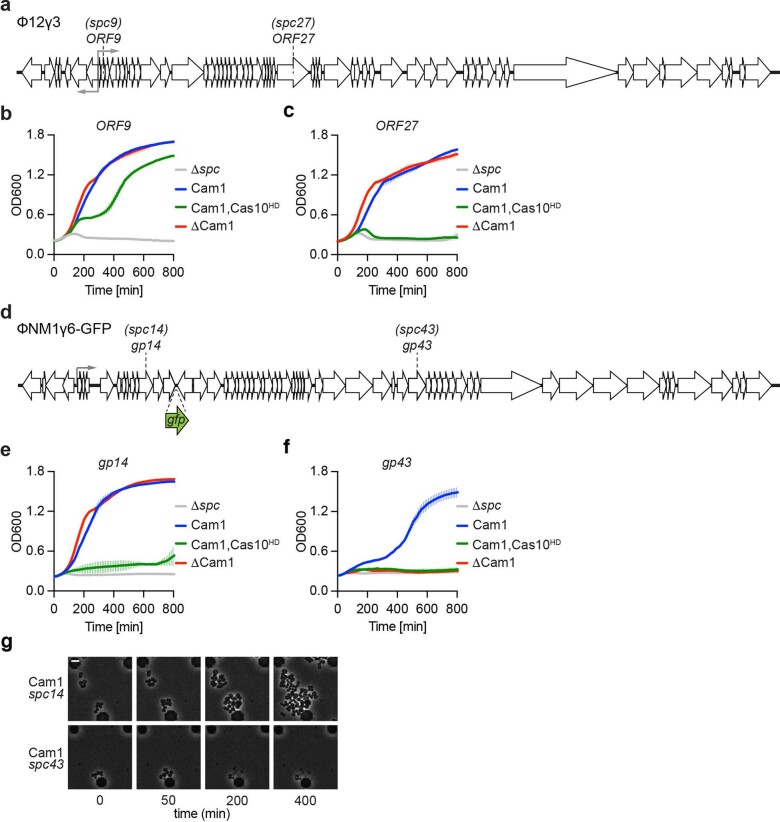
NhCam1 mediates phage defense. (**a**) Schematic of the genome of the staphylococcal phage Φ12γ3, showing the location of the transcripts targeted by the different spacers of type III-A CRISPR-Cas system. Grey arrows indicate promoters. (**b**) Growth of staphylococci carrying different pCRISPR constructs carrying active NhCam1 and/or Cas10, programmed to target the *ORF27* transcript of Φ12γ3, measured as OD_600_ at different times after infection, at an MOI ~ 0.1. Mean of three biological triplicates ± s.e.m. are reported. (**c**) Same as in (**b**) but targeting the *ORF29* transcript. Mean of three biological triplicates ± s.e.m. are reported. (**d**) Schematic of the genome of the staphylococcal phage ΦNM1γ6-GFP, showing the location of the transcripts targeted by the different spacers of type III-A CRISPR-Cas system, as well as the insertion site of the *gfp* gene. Grey arrows indicate promoters. (**e**) Growth of staphylococci carrying different pCRISPR constructs programmed to target the *gp14* transcript of ΦNM1γ6-GFP, measured as OD_600_ at different times after infection, at an MOI ~ 5. Mean of three biological triplicates ± s.e.m. are reported. (**f**) Same as in (**e**) but targeting the *gp43* transcript. Mean of three biological triplicates ± s.e.m. are reported. (**g**) Time-course microscopy of *S. aureus* harboring different pCRISPR constructs after infection with ΦNM1γ6-GFP. Images are representative of two biological replicates. Scale bar is 3.0 μM. Source Data

We next tested the role of Cam1 during the type III-A CRISPR–Cas response against phage infection. Given the different nature of this response depending on the timing of expression of the target RNA during the lytic cycle^28^, we designed two spacers (*spc9* and *spc27*) producing crRNAs complementary to an early- and late-expressed transcript from the staphylococcal phage ϕ12γ3^3^ (*ORF9* and *ORF27*, respectively; Extended Data Fig. 9a). In addition, since the type III-A defence relies on the function of both CARF effectors and the nuclease activity of Cas10^3,28^, we tested different mutant type III-A systems with (1) *cam1* and wild-type *cas10* (pCRISPR(*Nh*Cam1)); (2) with *cam1* but without the nuclease activity of *cas10* (pCRISPR (*Nh*Cam1, dCas10)); and (3) without *cam1* but with wild-type *cas10* (pCRISPR(ΔCam1)); as well as with a non-targeting spacer (Δ*spc*) as a negative (no immunity) control (Extended Data Fig. 1b). After infection at a multiplicity of infection (MOI) of approximately five, we observed that when the target RNA is recognized early in the viral lytic cycle (*spc9*), Cas10 alone can provide defence to support the continued growth of staphylococci, measured as the OD_600_ of the infected culture (Fig. 5a), in agreement with previous reports^3^. *Nh*Cam1 alone, however, provided much weaker immunity, which improved at a low MOI (around 0.1) (Extended Data Fig. 9b). Both Cas10- and *Nh*Cam1-mediated defence, alone and together, resulted in a significant decrease of the viral particle count of the cultures (two to three orders of magnitude reduction, measured as plaque-forming units (PFU); Fig. 5b). By contrast, when the type III-A response is delayed until late in the lytic cycle (*spc27*), both Cas10 and *Nh*Cam1, but not either of these on their own, are required to support the growth of the infected cells (Fig. 5c) and reduce the viral PFU count (Fig. 5d). *Nh*Cam1 did not provide defence against ϕ12γ3 infection by itself, even at the lower MOI of 0.1 (Extended Data Fig. 9c). We conclude that—as with previously described CARF effectors—*Nh*Cam1 is essential when the type III-A immune response is activated late in the viral lytic cycle, when the nuclease activity of Cas10 is not sufficient to control the infection.

The toxic effects of *Nh*Cam1 activation on the host cell and the observation that its efficiency is higher at lower MOIs are both hallmarks of abortive infection mechanisms of defence^41^. In this mode of immunity, infected cells do not survive infection (they either stop growing or lyse), they but do not allow for the propagation of the invading phage. As a result, uninfected cells in the culture can continue growing and the bacterial population survives viral predation. We next used microscopy to determine the fate of *Nh*Cam1-activated cells after infection with ϕNM1γ6-GFP, a derivative of phage ϕNM1γ6^42^ that expresses GFP early during infection (Extended Data Fig. 9d) that is subject to similar *Nh*Cam1-mediated, expression-dependent, immunity as ϕ12γ3 (Extended Data Figs. 1b and 9d–f). We then monitored individual staphylococci under the microscope after flowing around 10^7^ PFU µl^−1^ of ϕNM1γ6-GFP for 15 min. When full type III-A immunity (in cells equipped with both *Nh*Cam1 and Cas10) was triggered early in the viral cycle (*spc14*), few staphylococci turned green and they continued growing throughout the experiment (Fig. 5e and Supplementary Video 4). This is most probably owing to the direct attack of the phage DNA by the Cas10 nuclease, which most probably ‘cures’ the infected cells before GFP expression commences. By contrast, the majority of cells harbouring pCRISPR(*Nh*Cam1, dCas10) turned green and stopped proliferating (Fig. 5e and Supplementary Video 5), but did not lyse as in the case of the Δ*spc* control bacteria (Fig. 5e and Supplementary Video 6). This result indicates that *Nh*Cam1 mediates a growth arrest when its membrane depolarization is triggered by phage infection, which cannot immediately halt the viral lytic cycle (it does not affect GFP expression) but eventually leads to the prevention of phage propagation (Fig. 5b). We also flowed phage at a lower concentration (approximately 10^6^ PFU µl^−1^) to corroborate that fewer cells turn green and the non-fluorescent, uninfected, cells proliferate over time (Extended Data Fig. 9g and Supplementary Video 7). Together, these results demonstrate that membrane depolarization mediated by *Nh*Cam1 can provide an abortive infection mechanism of defence.

In cultures harbouring *spc43* in the presence of both Cas10 and *Nh*Cam1 activities, infected staphylococci expressed GFP and entered growth arrest, whereas some cells that did not emit green fluorescence (which were presumably not infected), were able to slowly resume growth (Fig. 5e and Supplementary Video 8). Therefore, when the type III-A immune response is triggered after the phage has completed many stages of the lytic cycle, both *Nh*Cam1 and Cas10 are required to mediate abortive infection. By contrast, cultures expressing *Nh*Cam1 but without the nuclease activity of Cas10 did not regrow (Fig. 5e and Supplementary Video 9), even at a low phage MOI when there are fewer infected hosts (Extended Data Fig. 9g and Supplementary Video 10). Cells expressed GFP and stopped growing, with some also undergoing lysis. This result suggests that *Nh*Cam1 alone, when targeting a late-expressed transcript, is unable to mediate abortive infection. Together, these results demonstrate an essential role for *Nh*Cam1 during type III-A CRISPR–Cas immunity when the invading phage is recognized late in the infection cycle.

## Discussion

The CARF effectors associated with type III-A CRISPR–Cas systems that have been experimentally characterized to date^1–5^ cause a growth arrest through the degradation of host nucleic acids. Here we investigated the structure and function of Cam1, a CARF effector that lacks a nuclease domain, but contains a TMH domain that promotes the formation of a tetrameric pore that is required to provide immunity through membrane depolarization and growth arrest of the infected cell (Supplementary Discussion). Although we studied Cam1 in the context of the type III-A CRISPR–Cas immune response of *S. aureus*, given the presence of this CARF effector in many different organisms (Extended Data Table 1), and the fact that most CRISPR–Cas loci are able to transfer horizontally between different species^43,44^ to provide defence without the need of host factors, we believe that our findings for Cam1, which were similar for three different homologues, would apply in the native hosts. Supporting this idea, we previously found that the Palm domain of the staphylococcal Cas10 subunit produces cA_6_ and cA_4_ to heterologously activate *Enterococcus italicus* Csm6^7^ and *Treponema succinifaciens* Card1^3^ in staphylococcal hosts.

During phage infection, the function of Cam1 is coupled with the type III-A CRISPR–Cas response, and therefore depolarization does not start until the Cas10 Palm domain synthesizes the cA_4_ ligand, which depends on the timing of target transcription during the viral lytic cycle. When Cam1 is activated early during infection, it arrests phage-infected cells, making them inviable for viral propagation. As the phages in the culture are ‘captured’ by the host, uninfected cells can continue growing. This mechanism provides effective immunity when the phage concentration is low and not all cells in the culture are infected. Thus, by causing membrane depolarization of the infected host, Cam1 mediates an abortive infection defence that protects the population rather individual bacterial cells. When the viral target is expressed late in the lytic cycle, the activation of Cam1 is insufficient to provide defence, even at a low multiplicity of infection. This is in contrast to other CARF effectors that cleave nucleic acids, such as Card1^3^ and Csm6^7^. We speculate that, although these effectors can directly affect viral replication through the degradation of phage RNA and/or DNA, Cam1-mediated depolarization affects the phage only indirectly, and therefore may not be able to interfere efficiently with the late phase of the viral cycle. Immunity is achieved only in conjunction with Cas10, which enables the regrowth of uninfected cells in the culture. We believe that the nuclease activity of Cas10 provides a direct attack on the phage DNA that complements the membrane depolarization caused by Cam1 to effectively inhibit the final stages of infection. Our results show the evolutionary flexibility of type III CRISPR–Cas systems, which have co-opted effectors with activities beyond nucleic acid degradation^45^ to mediate an abortive infection mechanism that protects the host when the cell-autonomous CRISPR response is not available.

## Methods

### Sequence alignments

Alignments and calculation of sequence identity and similarity (Extended Data Fig. 3a) were determined using EMBOSS Needle pairwise sequence alignment^46^.

### Bacterial growth

*S. aureus* strain RN4220^29^ was grown in brain heart infusion (BHI) medium at 37 °C, supplemented with chloramphenicol at 10 μg ml^−1^ for maintaining pCRISPR, and erythromycin at 10 μg ml^−1^ for maintaining pTarget. CaCl_2_ (5 μM) was supplemented in phage experiments unless indicated otherwise.

### Plasmid cloning

The plasmids used in this study are listed in Supplementary Table 1. The oligonucleotides used for this cloning are listed in Supplementary Table 2. The cloning strategies for generating these plasmids are listed in Supplementary Table 3. For obtaining the coding sequence of Cam1, the amino acid sequence of NCBI reference sequence WP_013033759.1 from *N. halophilus* Nc4 was synthesized by Genewiz.

### Growth curves

For in vivo Cam1 toxicity induction, triplicate RN4220 overnight cultures containing pTarget and pCRISPR were diluted 1:100, outgrown for about 1 h and normalized for optical density. Cells were then seeded in a 96-well plate. To induce targeting, 125 ng ml^−1^ of aTc was added to the appropriate wells. Absorbance at 600 nm was then measured every 10 min with a microplate reader (TECAN Infinite 200 PRO). All growth curve data were plotted using GraphPad Prism version 9.3.

For in vivo antiphage immunity, cells containing various pCRISPR constructs were launched in triplicate overnight, diluted 1:100, outgrown for about 1 h and normalized for optical density. Cells were seeded into a 96-well plate. Phage Φ12γ3^47^ or ΦNM1γ6^42^ was added at the specified MOIs, and optical density measurements were taken every 10 min.

### Cam1 toxicity assay

To measure the effect of Cam1 activity on *S. aureus* viability over time, colonies of *S. aureus* containing pTarget and the specified pCRISPR were launched in liquid culture overnight in triplicate. The next day, cells were diluted 1:100 and grown out for about 1 h and normalized for optical density. One aliquot was taken from each culture, and then aTc was added to induce CRISPR targeting and Cam1 activity. At each time point, cell aliquots were removed, centrifuged and resuspended in medium lacking aTc, and serial dilutions were plated on solid BHI agar plates with or without aTc. All viable cells should grow on the solid agar plates, but only targeting escapers (cells that recover owing to mutations in pTarget or pCRISPR) should form colony-forming units on plates with aTc.

### Quantification of phage plaques

To obtain PFU counts over time from cultures infected with phage, *S. aureus* cultures containing various pCRISPRs were launched overnight, diluted 1:100 and outgrown for about 1 h. Cells were then infected with phage Φ12γ3 at an MOI of 1, and an aliquot was taken shortly after to obtain plaque-forming units at time 0. The cultures were then incubated further, with aliquots taken at 1 h and 4 h.

### Cam1 toxicity time-course microscopy

To monitor the effects of Cam1 toxicity dynamics in greater detail, colonies of *S. aureus* containing pTarget and the specified pCRISPRs were launched in liquid culture overnight. The next day, cells were diluted 1:200 and were loaded into a CellASIC ONIX microfluidic plate along with medium containing plain BHI and medium containing BHI spiked with 125 ng ml^−1^ aTc. The plate was sealed and connected to a CellASIC ONIX2 Microfluidic System for microfluidic control of cells and media. Cells were incubated in the plate for 1 h at 37 °C using a Tokai HIT thermal box (Zeiss) until they were loaded onto the imaging chamber. Cells were imaged with phase contrast every 2 min for 8 h. Plain BHI was flowed over cells for the first 75 min followed by BHI spiked with aTc for the remaining 6 h 45 min. Imaging was performed in a Nikon Eclipse Ti2 motorized microscope with Perfect Focus System using a CFI60 Plan Apochromat Lambda Phase Contrast DM 100× Oil Immersion objective lens (Nikon) with a Zyla 4.2 sCMOS (Andor) camera (65 nm pixels). We used a SOLA Light Engine (Lumencor) as a laser source with laser power set to 20% with an exposure time of 10 ms. All medium was flown over cells with a constant pressure of 13.8 kPa. All microscopy data were analysed with NIS-Elements AR version 5.21.03 and Fiji version 2.3 software.

### Time-course fluorescence microscopy of phage-infected cultures

To visualize the dynamics of phage infection and immunity provided by Cam1, colonies of *S. aureus* containing various pCRISPRs with spacers programmed to target specified open reading frames in ϕNM1γ6-GFP were launched in liquid culture overnight. The next day, cells were diluted 1:200 and were loaded into a CellASIC ONIX microfluidic plate along with medium containing plain BHI supplemented with 2.5 mM CaCl_2_ with and without ϕNM1γ6-GFP at a titre of 3.32 × 10^7^ PFU μl^−1^. The plate was sealed and connected to a CellASIC ONIX2 Microfluidic System for microfluidic control of cells and media. Cells were incubated in the plate for 1 h at 37 °C using a Tokai HIT thermal box (Zeiss) until they were loaded onto the imaging chamber. Cells were imaged with phase contrast and in a GFP fluorescence channel every 2 min for 17 h. For the first hour, BHI supplemented with CaCl_2_ was flowed over cells followed by 15 min of phage flowed over. Finally, BHI supplemented with CaCl_2_ was flowed over for the remaining 15 h 45 min. The same phase contrast settings used in the Cam1 toxicity microscopy were used in these experiments, however, the GFP channel was measured with a C-FL GFP HC HISN Zero Shift filter (excitation: 470/40 nm (450–490 nm), emission: 525/50 nm (500–550 nm), dichroic mirror: 495 nm) (Nikon). GFP channel imaging was performed with the SOLA Light Engine set to 2% laser power with a 100 ms exposure time. All medium was flown over cells with a constant pressure of 13.8 kPa.

### Cell fractionation and western blotting

To assess the subcellular localization of Cam1, hexahistidine-tagged +Csm6 or +Cam1 pCRISPR RN4220 cultures were launched overnight. As a membrane protein control, an RN4220 culture harbouring pC194 with hexahistidine-tagged AgrB under the control of an IPTG-inducible promoter was also launched in the presence of 2 mM IPTG. All overnight cultures were diluted to an OD_600_ of 0.05 and were grown to an OD_600_ of 0.5 in the presence of 2 mM IPTG when appropriate. Cells were spun down at 3,900 rpm, decanted, and resuspended in lysis buffer (50 mM HEPES pH 7.0, 150 mM NaCl). Resuspended cultures were incubated with 2 mg ml^−1^ lysostaphin and cOmplete, Mini Protease Inhibitor Cocktail (Roche) for 15 min at 37 °C. The cultures were then sonicated and spun down at 3,900 rpm. An aliquot of these supernatants were taken as a whole cell lysate sample. These supernatants were then subjected to ultracentrifugation at 100,000*g* for 1 h. An aliquot of the supernatants was collected as a cytosolic fraction sample. The remaining supernatants were discarded, and the membrane pellets were resuspended with additional lysis buffer and were homogenized with a Teflon Dounce homogenizer. The homogenized pellets were subjected to another ultracentrifugation spin and supernatants were removed. The pellets were resuspended and homogenized again in lysis buffer and aliquots of these were collected as a membrane fraction samples. These proteins were run on 4–20% Mini-PROTEAN TGX Precast Protein Gels (Bio Rad). Transferred proteins were probed with THE His Tag anti-His_6_ antibodies in PBST (1:4,000 dilution) (GenScript, A00186) and blotting control RNA polymerase β was probed with anti-*E. coli* RNA polymerase β antibody in PBST (1:4,000 dilution) (BioLegend, 663903). Goat anti-rabbit IgG (H + L) Highly Cross-Adsorbed Secondary Antibody, HRP in PBST (1:10,000 dilution) (Invitrogen, A16110) was used to prepare the blots for imaging.

### Flow cytometry

For our membrane depolarization studies, colonies of *S. aureus* containing pTarget and the specified pCRISPR were launched in liquid culture overnight in triplicate. The next day, cells were diluted 1:100 and grown out for about 1 h and normalized to 10^7^ cells ml^−1^ in PBS. These cultures were then split into three different subcultures and treated with either 125 ng ml^−1^ aTc, 1.7 μM CCCP (Thermo Fisher), or nothing. These subcultures were incubated in shaking conditions at 37 °C for 30 min followed by addition of addition of 15 μM DiOC_2_(3) (Thermo Fisher) and incubation at room temperature for 30 min. Cells were then analysed on a BD LSR II (BD Biosciences) using BD FACSDiva software version 8.0.2 with 100,000 post-gating events recorded for each sample. Red/green ratios were calculated by using mean fluorescence intensities of all recorded events for each channel. The data were analysed with FlowJo v10.8.1. Gating strategy is shown in Supplementary Fig. 4.

### In vivo chemical crosslinking

To investigate Cam1 oligomerization in vivo, overnight cultures of *S. aureus* containing pTarget and pCRISPR +Cam1 was launched. These cultures were diluted 1:100, outgrown for about 1 h and normalized for optical density. Cultures were treated with 125 ng ml^−1^ aTc when appropriate and were incubated for a further 30 min. Cells were then spun down at 3,900 rpm and washed twice with 1× PBS pH 7.4 to remove free lysines and cysteines in the medium. Where indicated, cells were then treated with 2 mM DSS and BMH. Cells were then incubated with shaking at room temperature for 1 h. To quench the crosslinking reactions, cells were treated with 20 mM Tris-HCl pH 8.0 and 10 mM l-cysteine for 30 min shaking at room temperature. Quenched cultures were then digested with lysostaphin and sonicated. Cells were spun down and aliquots of the supernatant were collected for western blot analysis. Western blots were performed the same way they were in the fractionation experiments, except with no probing for Rpoβ.

### Protein expression and purification

The N-terminal truncated Cam1(42–206) or Cam1(66–206) were cloned to plasmid pRSF-Duet-1 with an N-terminal His_6_–SUMO tag followed by a Ulp1 cleavage site. The protein was overexpressed in *Escherichia coli* strain BL21 (DE3). Bacteria were grown at 37 °C to an OD_600_ of 0.6 and induced by 1 mM isopropyl β-d-1-thiogalactopyranoside (IPTG) at 16 °C overnight. Bacterial cells were lysed by sonication in lysis buffer (25 mM Tris-HCl, 500 mM NaCl, 5 mM 2-mercaptoethanol, pH 8.0) supplemented with 1 mM phenylmethylsulfonyl fluoride (PMSF). Cell lysates were centrifuged at 13,000*g* for 1 h, supernatants were loaded onto 5 ml HisTrap FF column (GE Healthcare) with extensive washing by lysis buffer supplemented with 40 mM imidazole. The target protein was eluted with lysis buffer supplemented with 400 mM imidazole, the elution was incubated with Ulp1 protease to cleave off the His_6_–SUMO tag during dialysis at 4 °C overnight against lysis buffer. The His_6_–SUMO tag was removed by using a HisTrap FF column (GE Healthcare) and target protein was further purified on Superdex 200 10/300 column pre-equilibrated in buffer A (25 mM Tris-HCl, 500 mM NaCl, 5 mM 2-mercaptoethanol, 5% glycerol, pH 8.0) for crystallization or in buffer B (25 mM Tris-HCl, 500 mM NaCl, 2 mM 2-mercaptoethanol, pH 8.0) for isothermal titration calorimetry experiments.

Full-length Cam1 with an N-terminal MGSS–His_6_–TEV site was overexpressed in *E. coli* cells in the presence of kanamycin (50 μg ml^−1^) and cells were induced with 0.2 mM IPTG as the OD_600_ reached 0.8–1. The cells were collected and resuspended in lysis buffer (25 mM HEPES pH 8, 500 mM NaCl, 2 mM β-mercaptoethanol, 5 % glycerol). The cells were broken using high-pressure homogenization and unlysed cells were separated by centrifugation at 14,000 rpm for 15 min at 4 °C. The cell membrane was purified by centrifugation at 28,000 rpm for 2 h at 4 °C. The purified membrane was hand homogenized, and the protein was extracted from the membrane using 1% DDM (Anatrace) containing lysis buffer. The insolubilized membrane was separated by centrifugation at 28,000 rpm for 1 h at 4 °C. The protein was purified from the supernatant using HisTrap FF column (Cytiva). The column was washed, and the protein was eluted with 25 mM HEPES pH 8, 500 mM NaCl, 2 mM β-mercaptoethanol, 5% glycerol and 0.05% DDM buffer supplemented with 40 mM and 300 mM imidazole respectively. The pure fractions of the protein were pooled and concentrated for the first round of size-exclusion chromatography (SEC) using a S200 increase column in an AKTA-Pure instrument. The buffer used for the SEC runs is 25 mM Tris pH 8, 500 mM NaCl, 2 mM β-mercaptoethanol and 0.02% DDM. The pure peak fractions were separated by SDS–PAGE and pooled for SEC–MALS analysis.

### Cam1 SEC–MALS analysis

The oligomeric state of full-length Cam1 protein (with theoretical molecular weight of a Cam1 monomer as 24.9 kDa) was determined by SEC–MALS conjugate analysis. The experiments were performed using an AKTA-Pure UV detector connected to SEC–MALS instrument with light scattering and refractive index detectors (Wyatt). The conversion factor between the digital UV signal of AKTA-Pure UV detector and analogue signal in SEC–MALS instrument is 1,000 mAU = 1 V. The SEC profile was analysed using ASTRA 6 software. For the conjugate analysis the refractive index increment (*dn*/*dc*) values for the Cam1 protein and detergent (DDM) were 0.185 ml g^−1^ and 0.1435 ml g^−1^, respectively.

### Crystallization and structure determination

As for apo Cam1(42–206), the protein was prepared at 13.8 mg ml^−1^ in buffer A. As for cA_4_–Cam1(42–206) or cA_6_–Cam1(42–206) complex, cA_4_ or cA_6_ at a final concentration of 1 mM was added to 13.8 mg ml^−1^ Cam1 in buffer A and incubated on ice for 1 h before crystallization. Crystallization conditions were determined with crystal screen (Qiagen) by sitting-drop vapour diffusion. Apo Cam1(42–206) crystals were grown from drops with 1.5 μl protein solution and 1.5 μl reservoir solution (0.2 M sodium chloride, 0.1 M sodium cacodylate trihydrate, pH 6.6, 25% PEG3350 (w/v)). cA_4_–Cam1(42–206) crystals were grown from drops with 1.5 μl protein solution and 1.5 μl reservoir solution (0.2 M ammonium sulfate, 0.1 M phosphate citrate, pH 4.2, 20% (w/v) PEG300, 10% glycerol). cA_6_–Cam1(42–206) crystals were grown from drops with 1.5 μl protein solution and 1.5 μl reservoir solution (0.05 M magnesium chloride hexahydrate, 0.1 M HEPES, pH 7.5, 30% (w/v) PEGMME 550). Crystals were cryoprotected using mother liquor containing 20% glycerol and flash-frozen in liquid nitrogen.

All diffraction data sets were collected on the 24-IE beamline at the Advanced Photon Source (APS) at the Argonne National Laboratory, and auto-processed by CDS package^48^ in the NE-CAT RAPD online server. The structures of apo, cA_4_- and cA_6_- bound Cam1(42–206) were solved by molecular replacement by using the structure predicted by AlphaFold^49^ as a search model. Iterative manual model building was performed using the program COOT^50^, and refinement with phenix.refine^51^ to produce the final models. The statistics of the data collection and refinement are shown in Supplementary Table 4. Figures were generated using PyMOL (http://www.pymol.org).

### Isothermal titration calorimetry

Wild-type and mutant Cam1(66–206) proteins were diluted to the final concentrations of 10 μM in buffer B and were titrated against 50 to 600 μM cyclic oligoadenylates in the same buffer at 20 °C by using MicroCal PEAQ-ITC range (Malvern Panalytical, HTRSC, Rockefeller University). The titration sequences included a single 0.5 μl injection, followed by 18 injections of 2 μl each, with 2 min intervals between injections and a stirring rate of 1,000 rpm. Calorimetric data were analysed using OriginLab software (GE Healthcare) and AFFINmeter web-based software (www.affinimeter.com), and final graphs were represented by Origin version 7.0.

### Reporting summary

Further information on research design is available in the Nature Portfolio Reporting Summary linked to this article.

## Online content

Any methods, additional references, Nature Portfolio reporting summaries, source data, extended data, supplementary information, acknowledgements, peer review information; details of author contributions and competing interests; and statements of data and code availability are available at 10.1038/s41586-023-06902-y.

### Supplementary information

Supplementary Discussion

Reporting Summary

Supplementary Fig. 1 Raw images. (a) Fig. 3c; (b) Fig. 4a, inverted, molecular weight ladder included; (c) Fig. 4e and 4f; (d) Extended Data Fig. 1b; (e) Extended Data Fig. 6d; (f) Extended Data Fig. 6f; (g–o) Extended Data Fig. 6b.

Supplementary Fig. 2 Comparison of *Nh*Cam1-CARF to other dimeric CARF domains that bind cA4. (a) Comparison of the overall topology of dimeric CARF domains bound to cA4. (b) Hydrogen bonds between the dimeric CARF domain and cA4 in different CARF effectors. (c) Same as (b) but showing hydrophobic interactions.

Supplementary Fig. 3 Stabilizing dimeric interface for the binding cA_4_. Atoms that make this interface are shown in grey ball and stick models for *Nh*Cam1, ToCsm6, SiCsx1 and TsCard1. The area of this interface is noted.

Supplementary Fig. 4 Gating strategy for depolarization flow cytometry. (a) Gating strategy schematic for depolarization analysis. (b) Example of cell populations after each step within the gating strategy.

Supplementary Table 1 Plasmids used in this study.

Supplementary Table 2 Oligonucleotide primers used in this study.

Supplementary Table 3 Cloning strategies used in this study.

Supplementary Table 4 Structural data collection and refinement statistics.

Supplementary Video 1 Live microscopy of staphylococci harbouring pTarget and pCRISPR(D*spc*) after addition of aTc. Representative sections of the field of view at different times after addition of aTc were shown in Fig. 1d.

Supplementary Video 2 Live microscopy of staphylococci harbouring pTarget and pCRISPR(*Nh*Cam1) after addition of aTc. Representative sections of the field of view at different times after addition of aTc were shown in Fig. 1d.

Supplementary Video 3 Binding of cA_4_. The movie shows a model of the structural transition of the C-terminal b strand b7 and loop L2 on proceeding from the structure of the CARF domain of *Nh*Cam1 in the apo state to the cA_4_ bound state.

Supplementary Video 4 Live microscopy of staphylococci harbouring pCRISPR(*Nh*Cam1-*spc14*) after infection with fNM1g6–GFP. ~10^7^ PFU µl^−1^ of phage were flowed for 15 min. Representative sections of the field of view at different times after addition of phage were shown in Fig. 4e.

Supplementary Video 5 Live microscopy of staphylococci harbouring pCRISPR(D*spc*) after infection with fNM1g6–GFP. ~10^7^ PFU µl^−1^ of phage were flowed for 15 min. Representative sections of the field of view at different times after addition of phage were shown in Fig. 4e.

Supplementary Video 6 Live microscopy of staphylococci harbouring pCRISPR(*Nh*Cam1,dCas10- *spc14*) after infection with fNM1g6–GFP. ~10^7^ PFU µl^−1^ of phage were flowed for 15 min. Representative sections of the field of view at different times after addition of phage were shown in Fig. 4e.

Supplementary Video 7 Live microscopy of staphylococci harbouring pCRISPR(*Nh*Cam1,dCas10- *spc14*) after infection with fNM1g6–GFP. ~10^6^ PFU µl^−1^ of phage were flowed for 15 min. Representative sections of the field of view at different times after addition of phage were shown in Extended Data Fig. 7g.

Supplementary Video 8 Live microscopy of staphylococci harbouring pCRISPR(*Nh*Cam1-*spc43*) after infection with fNM1g6–GFP. ~10^7^ PFU µl^−1^ of phage were flowed for 15 min. Representative sections of the field of view at different times after addition of phage were shown in Fig. 4e.

Supplementary Video 9 Live microscopy of staphylococci harbouring pCRISPR(*Nh*Cam1,dCas10- *spc43*) after infection with fNM1g6–GFP. ~10^7^ PFU µl^−1^ of phage were flowed for 15 min. Representative sections of the field of view at different times after addition of phage were shown in Fig. 4e.

Supplementary Video 10 Live microscopy of staphylococci harbouring pCRISPR(*Nh*Cam1,dCas10- *spc43*) after infection with fNM1g6–GFP. ~10^6^ PFU µl^−1^ of phage were flowed for 15 min. Representative sections of the field of view at different times after addition of phage were shown in Extended Data Fig. 7g.

### Source data

Source Data Fig. 1

Source Data Fig. 2

Source Data Fig. 3

Source Data Fig. 4

Source Data Fig. 5

Source Data Extended Data Fig. 1

Source Data Extended Data Fig. 2

Source Data Extended Data Fig. 3

Source Data Extended Data Fig. 4

Source Data Extended Data Fig. 6

Source Data Extended Data Fig. 7

Source Data Extended Data Fig. 8

Source Data Extended Data Fig. 9

## Figures and Tables

**Extended Data Table 1:**
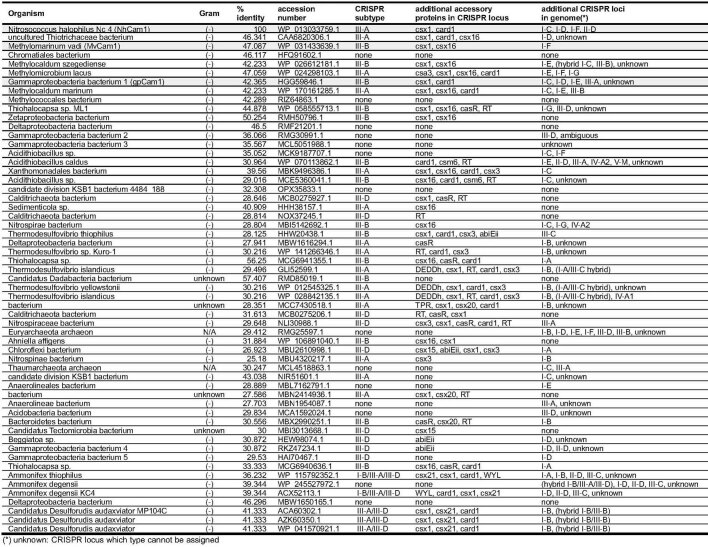
Cam1 homologs in prokaryotic genomes

## Data Availability

Atomic coordinates have been deposited in the Protein Data Bank with the accession codes 8T64 (apo *Nh*Cam1-CARF), 8T65 (cA_4_–*Nh*Cam1-CARF complex) and 8T66 (cA_6_–*Nh*Cam1-CARF complex). Cam1 escaper plasmid sequencing data have been deposited at NCBI with BioProject ID PRJNA1030403. Flow cytometry data have been deposited at FlowRepository with repository ID FR-FCM-Z7ZK. Source data are provided with this paper.
