# Supplementary material for: The CRISPR effector Cam1 mediates membrane depolarization for phage defence
Source: Nature. 2024 Jan 10;625(7996):797–804. doi: 10.1038/s41586-023-06902-y (PMC10808066; doi:10.1038/s41586-023-06902-y)
Supplement: Supplementary file 1 — Supplementary Discussion [file 41586_2023_6902_MOESM1_ESM.docx]

**Supplementary Discussion**

*Cam1 activation via cA_4_ binding*

Our biochemical experiments and crystallographic structures demonstrate that cA_4_ is the second messenger that targets the CARF domain dimer of NhCam1. Complex formation is mediated by a conformational change, whereby the bound cA_4_ is positioned between monomers and encapsulated by loop L2 elements through a capping process. The bound cA_4_ adopts *anti* glycosidic torsion angles at all four adenosines, with adenosine specificity attributed to hydrogen bonding recognition of a pair of adenosines on complex formation. The bound cA_4_ is anchored in place through a network of hydrogen bonding and hydrophobic interactions, with the importance of individual contributions to *in vitro* binding and *in vivo* function, elucidated from mutation studies. Notably, NhCam1 does not cleave bound cA_4_ to ApAp>, as has been reported previously for Csm6^21^, whereby the latter functions as a ring nuclease. Considering that this CARF effector is associated with the bacterial membrane, is likely to form a tetrameric transmembrane pore lined by aspartate residues, and mediates membrane depolarization, we hypothesize that cA_4_ binding results in a conformational change that facilitates ion movement across the cytoplasmic membrane. We looked for potassium release (using IPG-4 TMA^1^), however we were unable to detect significant signal changes after inducing target transcription (data not shown). Therefore, the nature of the depolarization mediated by Cam1 remains unknown.

*Evolutionary benefits of abortive infection*

Although Cam1 mechanism of defense has the drawback that cannot provide protection at high multiplicity of infection, when all cells of the bacterial culture are infected and cease to grow, it has the advantage of preventing the escape of phages carrying mutations in the target region that prevent recognition by the crRNA. Given that these mutant phages are infrequent, the majority of the bacteria will be infected with wild-type phages that activate Cam1-medaited membrane depolarization. Therefore, the mutant phages eventually end up entering a cell previously infected by wild-type phage that cannot support their propagation, and are eliminated from the culture^2^. This is in contrast to the CRISPR systems that provide cell autonomous immunity, for which phage escapers can propagate unchallenged^3^, and could one of the reasons for the evolutionary conservation of Cam1, other CARF effectors, and abortive infection systems in general.

*Other mechanisms related to Cam1 defense*

Cam1 function is similar to that of Csx28, a type VI CRISPR accessory protein that forms an octameric pore on the membrane^4^, as well as other effectors that cause membrane depolarization through a general disruption of membrane function rather than pore formation. These include Aga2, a membrane protein that associates with prokaryotic Argonaute^5^; and a group of CBASS effectors containing transmembrane helices, Cap14, Cap15 and Cap16, that upon binding of a cyclic nucleotide second messenger oligomerize to disrupt the membrane of infected cells^6^. Of these, Cap14 contains a SAVED domain, which is a distant homolog CARF domains that use cyclic oligoadenylate ligands^7^. Cam1 could also play a specific role in the defense against phages that utilize pinholins to lyse Gram-negative hosts. Phage produce pinholins to depolarize the bacterial membrane, cause collapse of the proton motive force, and activate phage endolysins to attack the host peptidoglycan^8,9^. In this context, Cam1-mediated depolarization could prematurely activate phage endolysins and host lysis.

*Cam1 co-occurrence with other immune effectors*

We found that most Cam1 homologs co-occur with other CARF effectors (Extended Data Table1 and Extended Data Fig. 8a), which could be necessary to complement Cam1 in the case of type III CRISPR systems that encode Cas10 enzymes that lack nuclease activity^10^. Given that CRISPR systems move horizontally across different bacterial species^11,12^, additional CARF effectors (or additional anti-phage defense loci, including CRISPR-Cas systems of different types) may also be necessary when the membrane composition of the host impairs pore formation and/or depolarization.

**References**

1 Prindle, A. *et al.* Ion channels enable electrical communication in bacterial communities. *Nature* **527**, 59-63, (2015).

2 Meeske, A. J., Nakandakari-Higa, S. & Marraffini, L. A. Cas13-induced cellular dormancy prevents the rise of CRISPR-resistant bacteriophage. *Nature* **570**, 241-245, (2019).

3 Barrangou, R. *et al.* CRISPR provides acquired resistance against viruses in prokaryotes. *Science* **315**, 1709-1712, (2007).

4 VanderWal, A. R. *et al.* Csx28 is a membrane pore that enhances CRISPR-Cas13b-dependent antiphage defense. *Science* **380**, 410-415, (2023).

5 Zeng, Z. *et al.* A short prokaryotic Argonaute activates membrane effector to confer antiviral defense. *Cell Host Microbe* **30**, 930-943 e936, (2022).

6 Duncan-Lowey, B., McNamara-Bordewick, N. K., Tal, N., Sorek, R. & Kranzusch, P. J. Effector-mediated membrane disruption controls cell death in CBASS antiphage defense. *Mol. Cell* **81**, 5039-5051, (2021).

7 Lowey, B. *et al.* CBASS Immunity Uses CARF-Related Effectors to Sense 3'-5'- and 2'-5'-Linked Cyclic Oligonucleotide Signals and Protect Bacteria from Phage Infection. *Cell* **182**, 38-49 e17, (2020).

8 Park, T., Struck, D. K., Dankenbring, C. A. & Young, R. The pinholin of lambdoid phage 21: control of lysis by membrane depolarization. *J. Bacteriol.* **189**, 9135-9139, (2007).

9 Young, R. Phage lysis: three steps, three choices, one outcome. *J Microbiol* **52**, 243-258, (2014).

10 Gruschow, S., Adamson, C. S. & White, M. F. Specificity and sensitivity of an RNA targeting type III CRISPR complex coupled with a NucC endonuclease effector. *Nucleic Acids Res.* **49**, 13122-13134, (2021).

11 Chakraborty, S. *et al.* Comparative network clustering of direct repeats (DRs) and cas genes confirms the possibility of the horizontal transfer of CRISPR locus among bacteria. *Mol. Phylogenet. Evol.* **56**, 878-887, (2010).

12 Godde, J. S. & Bickerton, A. The repetitive DNA elements called CRISPRs and their associated genes: evidence of horizontal transfer among prokaryotes. *J. Mol. Evol.* **62**, 718-729, (2006).
