# Supplementary material for: The CRISPR effector Cam1 mediates membrane depolarization for phage defence
Source: Nature. 2024 Jan 10;625(7996):797–804. doi: 10.1038/s41586-023-06902-y (PMC10808066; doi:10.1038/s41586-023-06902-y)
Supplement: Supplementary file 2 — Reporting Summary [file 41586_2023_6902_MOESM2_ESM.pdf]

nature portfolio

Corresponding author(s): Luciano Marraffini

Last updated by author(s): Oct 23, 2023

Reporting Summary

Nature Portfolio wishes to improve the reproducibility of the work that we publish. This form provides structure for consistency and transparency in reporting. For further information on Nature Portfolio policies, see our Editorial Policies and the Editorial Policy Checklist.

Statistics

For all statistical analyses, confirm that the following items are present in the figure legend, table legend, main text, or Methods section.

n/a	Confirmed	
☐	☒	The exact sample size (*n*) for each experimental group/condition, given as a discrete number and unit of measurement
☐	☒	A statement on whether measurements were taken from distinct samples or whether the same sample was measured repeatedly
☐	☒	The statistical test(s) used AND whether they are one- or two-sided<br>*Only common tests should be described solely by name; describe more complex techniques in the Methods section.*
☒	☐	A description of all covariates tested
☒	☐	A description of any assumptions or corrections, such as tests of normality and adjustment for multiple comparisons
☐	☒	A full description of the statistical parameters including central tendency (e.g. means) or other basic estimates (e.g. regression coefficient) AND variation (e.g. standard deviation) or associated estimates of uncertainty (e.g. confidence intervals)
☐	☒	For null hypothesis testing, the test statistic (e.g. *F*, *t*, *r*) with confidence intervals, effect sizes, degrees of freedom and *P* value noted<br>*Give P values as exact values whenever suitable.*
☒	☐	For Bayesian analysis, information on the choice of priors and Markov chain Monte Carlo settings
☒	☐	For hierarchical and complex designs, identification of the appropriate level for tests and full reporting of outcomes
☒	☐	Estimates of effect sizes (e.g. Cohen's *d*, Pearson's *r*), indicating how they were calculated

Our web collection on statistics for biologists contains articles on many of the points above.

Software and code

Policy information about availability of computer code

Data collection	CrisprCasTyper web server version 1.6.4
Data analysis	GraphPad Prism version 9.3<br>FlowJo version 10.8.1<br>PyMol version 2.5.2<br>Fiji version 2.3<br>NIS-Elements AR version 5.21.03<br>BD FACSDiva software version 8.0.2

For manuscripts utilizing custom algorithms or software that are central to the research but not yet described in published literature, software must be made available to editors and reviewers. We strongly encourage code deposition in a community repository (e.g. GitHub). See the Nature Portfolio guidelines for submitting code & software for further information.

Data

Policy information about availability of data

All manuscripts must include a data availability statement. This statement should provide the following information, where applicable:

- Accession codes, unique identifiers, or web links for publicly available datasets
- A description of any restrictions on data availability
- For clinical datasets or third party data, please ensure that the statement adheres to our policy

The atomic coordinates have been deposited in the Protein Data Bank with the codes 8CSK (apo Cam1-CARF), 8CSM (cA4-Cam1-CARF complex), and 8CSN (cA6-Cam1-CARF complex). PDB reports were provided as separate files. Cam1 escaper plasmid sequencing data is deposited on NCBI with BioProject ID: PRJNA1030403. Flow cytometry data is deposited in FlowRepository with repository ID: FR-FCM-Z7ZK.

Human research participants

Policy information about studies involving human research participants and Sex and Gender in Research.

Reporting on sex and gender	N/A
Population characteristics	N/A
Recruitment	N/A
Ethics oversight	N/A

Note that full information on the approval of the study protocol must also be provided in the manuscript.

Field-specific reporting

Please select the one below that is the best fit for your research. If you are not sure, read the appropriate sections before making your selection.

☒ Life sciences ☐ Behavioural & social sciences ☐ Ecological, evolutionary & environmental sciences

For a reference copy of the document with all sections, see nature.com/documents/nr-reporting-summary-flat.pdf

Life sciences study design

All studies must disclose on these points even when the disclosure is negative.

Sample size	Standard 3 biological replicates were used unless indicated otherwise.
Data exclusions	No data was excluded from the analysis
Replication	All experimental findings were reliably replicated as indicated in figure legends
Randomization	Not relevant to this study as there are no animal nor human experiments, and the experimental outcome does not depend on the order in which samples were analyzed in the experiments
Blinding	Not relevant to this study as there are no animal nor human experiments, and the knowledge the order or identity of a sample does not change the experimental outcome

Reporting for specific materials, systems and methods

We require information from authors about some types of materials, experimental systems and methods used in many studies. Here, indicate whether each material, system or method listed is relevant to your study. If you are not sure if a list item applies to your research, read the appropriate section before selecting a response.

Materials & experimental systems

n/a	Involved in the study
☐	☒ Antibodies
☒	☐ Eukaryotic cell lines
☒	☐ Palaeontology and archaeology
☒	☐ Animals and other organisms
☒	☐ Clinical data
☒	☐ Dual use research of concern

Methods

n/a	Involved in the study
☒	☐ ChIP-seq
☐	☒ Flow cytometry
☒	☐ MRI-based neuroimaging

Antibodies

Antibodies used

THE™ His Tag Antibody, mAb, Mouse - GenScript - catalog #A00186
Anti-E. coli RNA Polymerase β Antibody - BioLegend - catalog #663903
Goat anti-Rabbit IgG (H+L) Highly Cross-Adsorbed Secondary Antibody, HRP - Invitrogen - catalog #A16110

Validation

THE™ His Tag Antibody, mAb, Mouse - https://www.genscript.com/antibody/A00186-THE_His_Tag_Antibody_mAb_Mouse.html
Anti-E. coli RNA Polymerase β Antibody - https://www.biolegend.com/de-de/products/anti-e-coli-rna-polymerase-beta-antibody-10494
Goat anti-Rabbit IgG (H+L) Highly Cross-Adsorbed Secondary Antibody, HRP - https://www.thermofisher.com/antibody/product/Goat-anti-Rabbit-IgG-H-L-Highly-Cross-Adsorbed-Secondary-Antibody-Polyclonal/A16110

Flow Cytometry

Plots

Confirm that:

☒ The axis labels state the marker and fluorochrome used (e.g. CD4-FITC).

☒ The axis scales are clearly visible. Include numbers along axes only for bottom left plot of group (a 'group' is an analysis of identical markers).

☒ All plots are contour plots with outliers or pseudocolor plots.

☒ A numerical value for number of cells or percentage (with statistics) is provided.

Methodology

Sample preparation

Colonies of S. aureus containing pTarget and the specified pCRISPR were launched in liquid culture overnight in triplicate. The next day, cells were diluted 1:100 and grown out for about an hour and normalized to 107 cells ml-1 in PBS. These cultures were then split into three different subcultures and treated with either 125 ng ml-1 aTc, 1.7 µM carbonyl cyanide 3-chlorophenylhydrazone (CCCP) (Thermo Fisher), or nothing. These subcultures were incubated in shaking conditions at 37 °C for 30 minutes followed by addition of addition of 15 µM 3,3′- diethyloxacarbocyanine iodide (DiOC2(3)) (Thermo Fisher) and incubation at room temperature for 30 minutes. Cells were then analyzed on a BD® LSR II (BD Biosciences) with 100,000 events recorded for each sample. Red/Green ratios were calculated by using mean fluorescence intensities of all recorded events for each channel. The data was analyzed with FlowJo v10.8.1.

Instrument

BD-LSRII

Software

Collection - BD FACSDiva software version 8.0.2
Analysis -FlowJo version 10.8.1

Cell population abundance

N/A

Gating strategy

For analysis, all staphylococci were gated based on SSC-H vs SSC-A for singlets. A more detailed strategy is included in Supplementary Fig. 4.

☒ Tick this box to confirm that a figure exemplifying the gating strategy is provided in the Supplementary Information.
