# Supplementary figures and images for: The CRISPR effector Cam1 mediates membrane depolarization for phage defence
Source: Nature. 2024 Jan 10;625(7996):797–804. doi: 10.1038/s41586-023-06902-y (PMC10808066; doi:10.1038/s41586-023-06902-y)

**a**

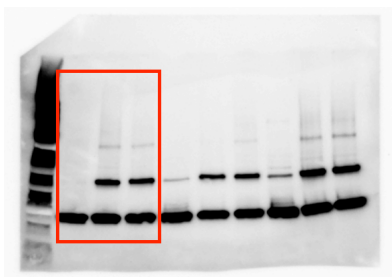

**b**

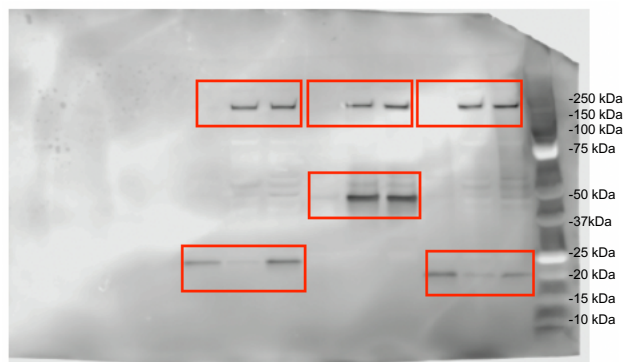

**c**

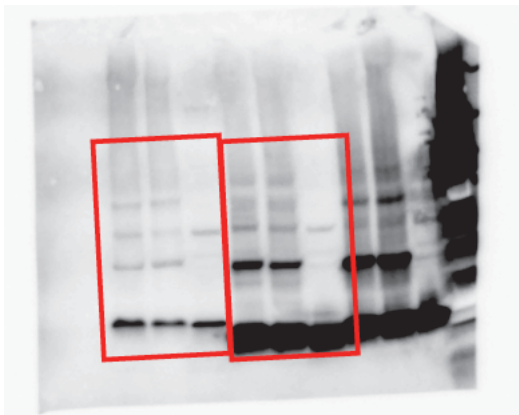

**d**

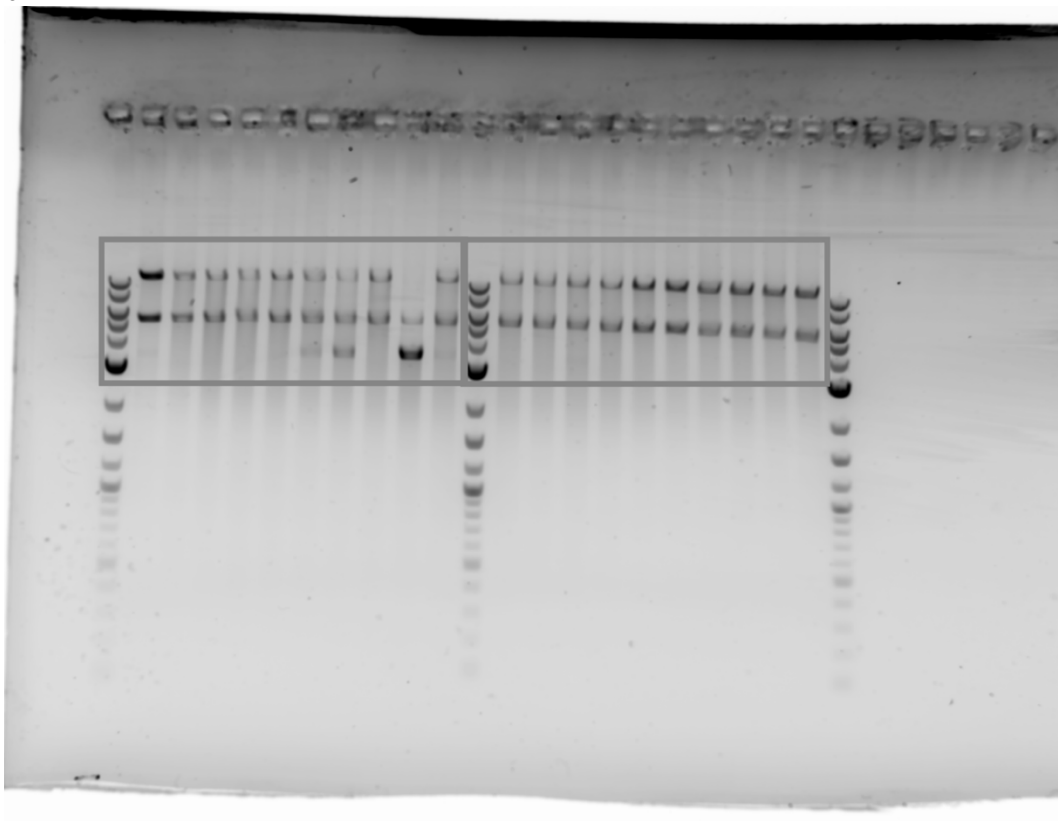

**e**

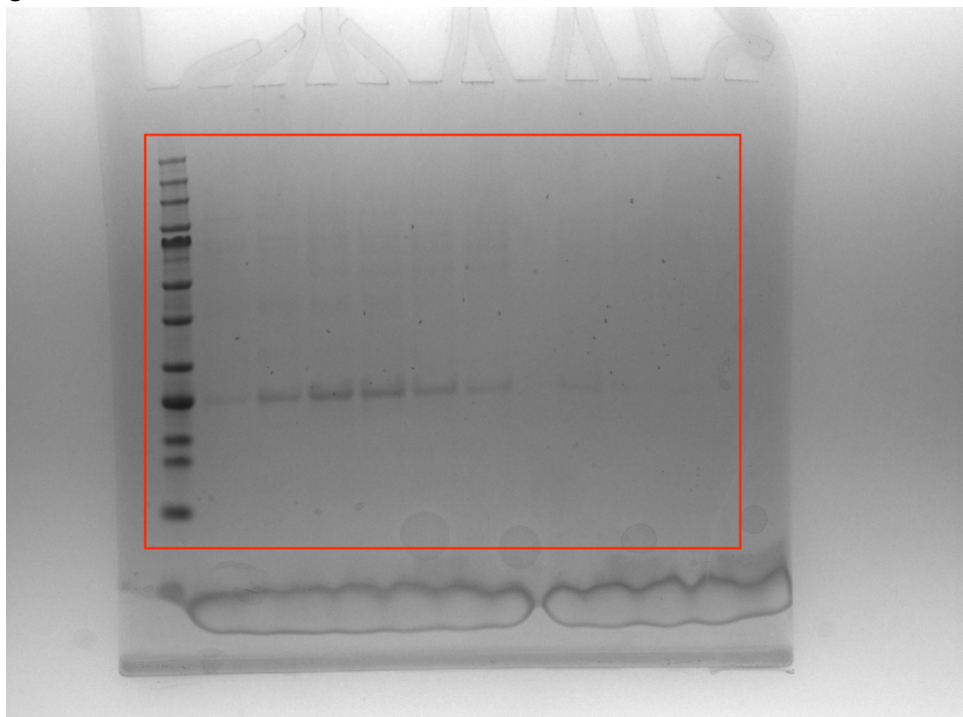

**f**

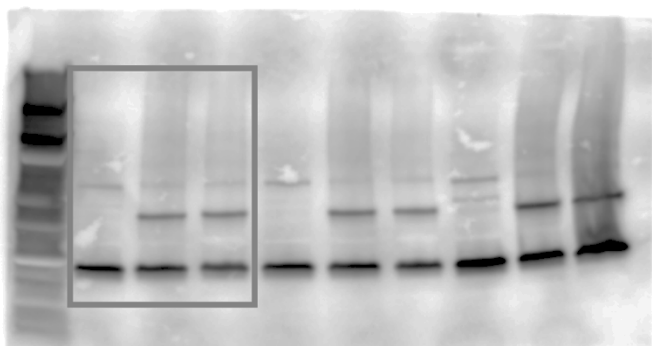

**g**

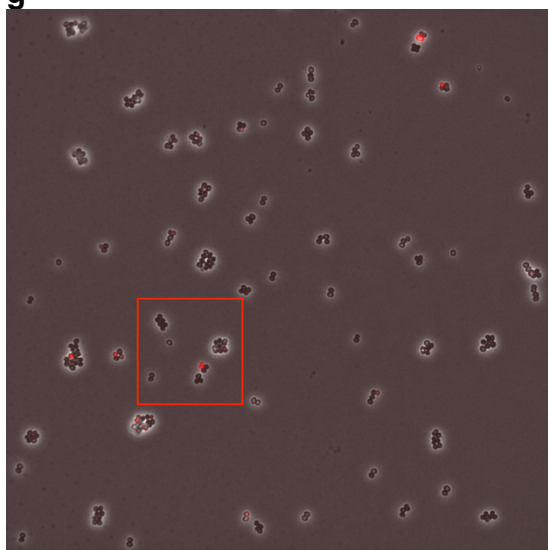

**h**

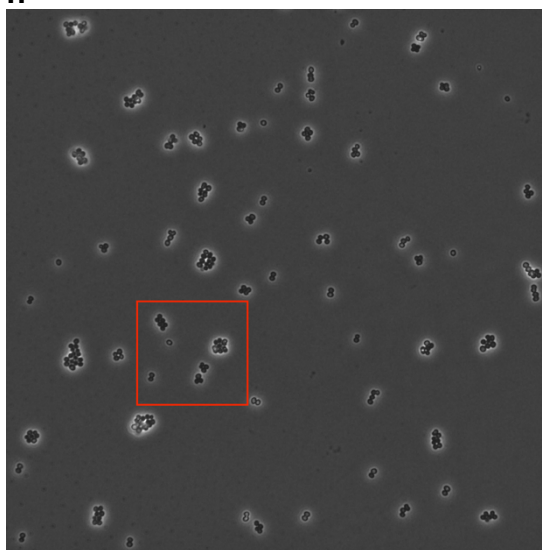

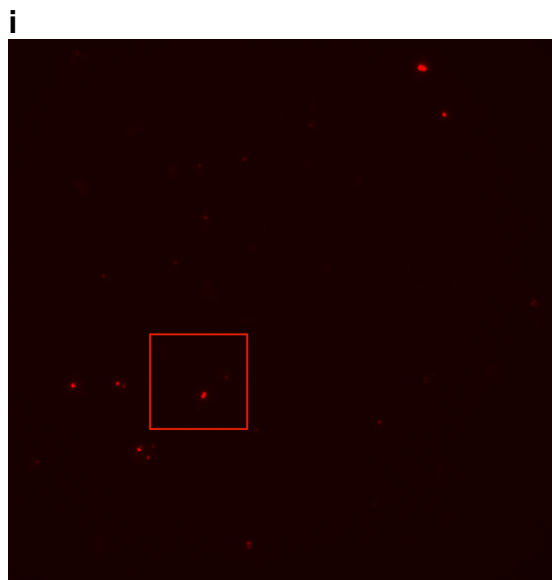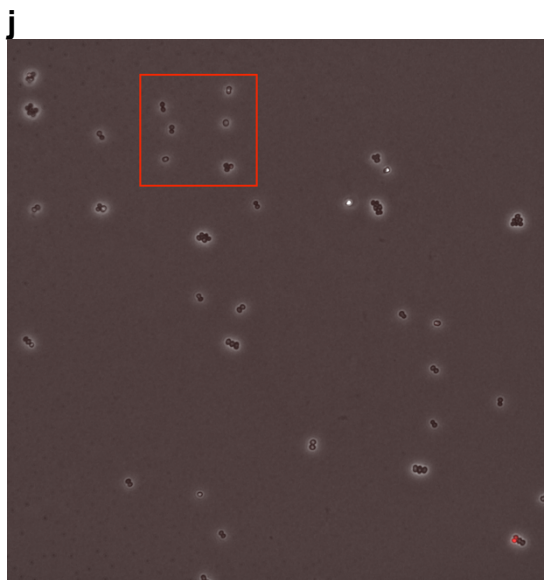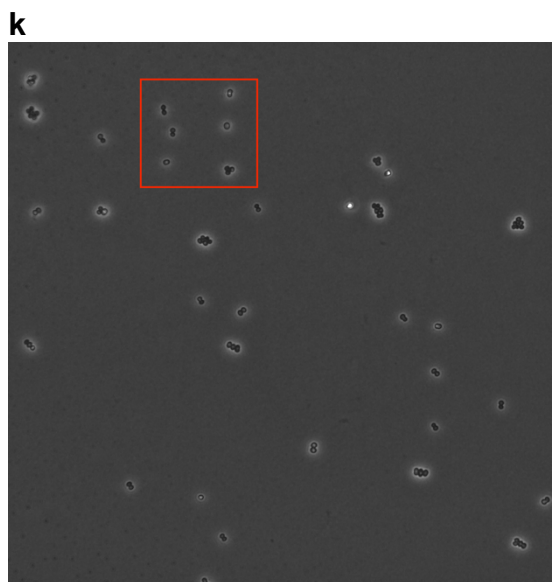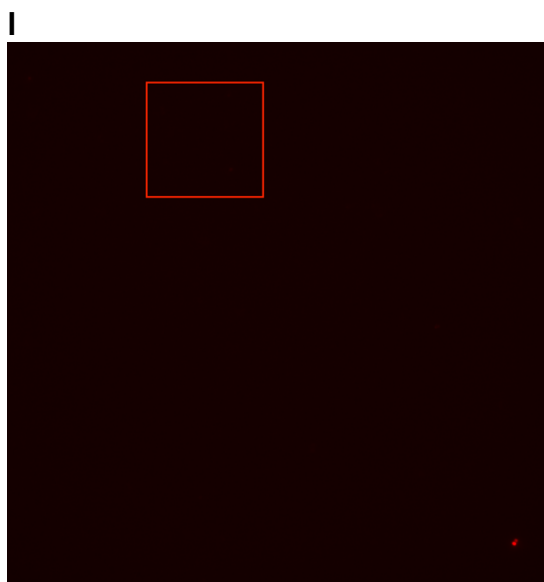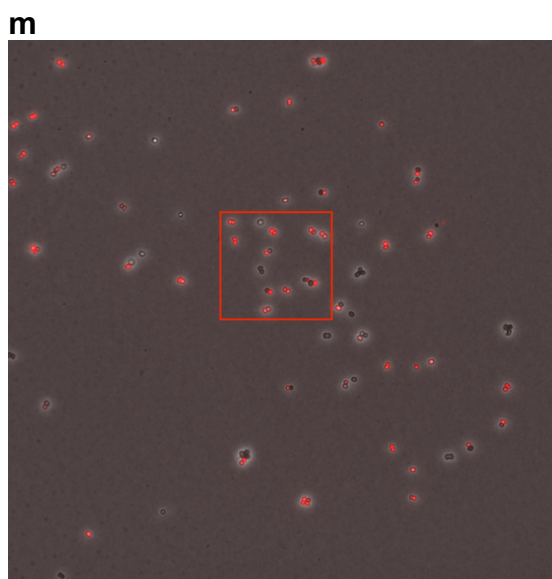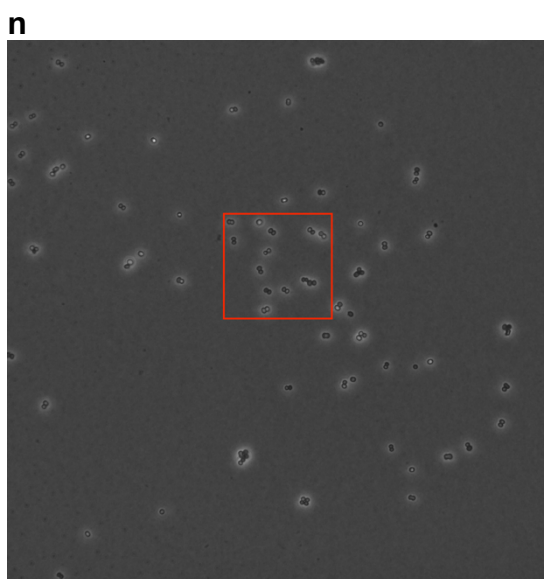

o

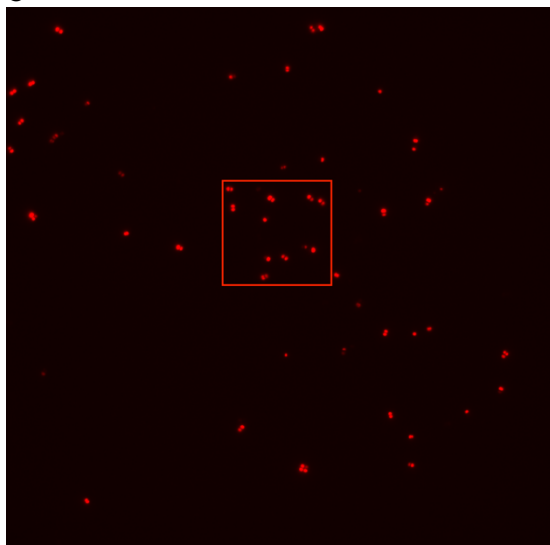

Supplement: Supplementary file 3 — Raw images. (a) Fig. 3c; (b) Fig. 4a, inverted, molecular weight ladder included; (c) Fig. 4e and 4f; (d) Extended Data Fig. 1b; (e) Extended Data Fig. 6d; (f) Extended Data Fig. 6f; (g–o) Extended Data Fig. 6b. [file 41586_2023_6902_MOESM3_ESM.pdf]
