# Supplementary material for: The CRISPR effector Cam1 mediates membrane depolarization for phage defence
Source: Nature. 2024 Jan 10;625(7996):797–804. doi: 10.1038/s41586-023-06902-y (PMC10808066; doi:10.1038/s41586-023-06902-y)
Supplement: Supplementary file 7 — Plasmids used in this study. [file 41586_2023_6902_MOESM7_ESM.docx]

**Supplementary Table 1.** Plasmids used in this study

| **Plasmid name** | **Plasmid contents** | **Made in this study?** |
| --- | --- | --- |
| pCFB1 | IPTG-inducible AgrB-Cterm-6xHis | Yes |
| pCFB2 | Type III, gp43 spacer, no accessory protein | Yes |
| pCFB5 | Type III, ORF27 spacer, no accessory protein | Yes |
| pCFB6 | Type III, ORF9 spacer, no accessory protein | Yes |
| pCFB20 | Type III Cas10HD/PALM mutant, gp43 spacer, Nh Cam1 | Yes |
| pCFB25 | Type III, gp14 spacer, no accessory protein | Yes |
| pCFB26 | Type III Cas10HD mutant, gp43 spacer, Nh Cam1 S75A + N76A | Yes |
| pCFB27 | Type III Cas10HD mutant, gp43 spacer, Nh Cam1 V79A | Yes |
| pCFB28 | Type III Cas10HD mutant, gp43 spacer, Nh Cam1 T97A | Yes |
| pCFB29 | Type III Cas10HD mutant, gp43 spacer, Nh Cam1 Y180A + T183A | Yes |
| pCFB31 | Type III Cas10HD mutant, gp43 spacer, Nh Cam1 N76A | Yes |
| pCFB32 | Type III Cas10HD mutant, gp43 spacer, Nh Cam1 S75A | Yes |
| pCFB33 | Type III Cas10HD mutant, gp43 spacer, Nh Cam1 Y180A | Yes |
| pCFB34 | Type III Cas10HD mutant, gp43 spacer, Nh Cam1 T183A | Yes |
| pCFB55 | GFP-NM1 homology recombination plasmid | Yes |
| pCFB58 | pCas9 with spacer to select for recombined GFP-NM1 | Yes |
| pCFB64 | Type III Cas10HD mutant, gp43 spacer, Nh Cam1 M194A | Yes |
| pCFB65 | Type III Cas10HD mutant, gp43 spacer, Nh Cam1 L189A | Yes |
| pCFB67 | Type III Cas10HD mutant, gp43 spacer, Nh Cam1 K160A | Yes |
| pCFB68 | Type III Cas10HD mutant, gp43 spacer, Nh Cam1 F185A | Yes |
| pCFB71 | Type III Cas10HD mutant, gp43 spacer, Nh Cam1 V79A + P192A + M194A | Yes |
| pCFB72 | Type III Cas10HD mutant, gp43 spacer, Nh Cam1 P192A | Yes |
| pCFB73 | Type III Cas10HD mutant, gp43 spacer, Nh Cam1 K160A + L189A + F185A | Yes |
| pCFB92 | Type III Cas10HD mutant, gp43 spacer, Nh Cam1 S24A | Yes |
| pCFB93 | Type III Cas10HD mutant, gp43 spacer, Nh Cam1 D17S | Yes |
| pCFB95 | Type III Cas10HD mutant, gp43 spacer, Nh Cam1 D17A | Yes |
| pCFB96 | Type III Cas10HD mutant, gp43 spacer, Nh Cam1 S24K | Yes |
| pCFB98 | Type III Cas10HD mutant, gp43 spacer, Nh Cam1 D17E | Yes |
| pCFB99 | Type III Cas10HD mutant, gp43 spacer, Nh Cam1 D17R | Yes |
| pCFB100 | Type III Cas10HD mutant, gp43 spacer, Nh Cam1 S24R | Yes |
| pCFB101 | Type III Cas10HD mutant, gp43 spacer, Nh Cam1 D17N | Yes |
| pCFB102 | Type III Cas10HD mutant, gp43 spacer, Nh Cam1 S24M | Yes |
| pCFB103 | Type III Cas10HD mutant, gp43 spacer, Nh Cam1 D17K | Yes |
| pCFB104 | Type III Cas10HD mutant, gp43 spacer, Nh Cam1 S24D | Yes |
| pCFB114 | Type III Cas10HD mutant, gp43 spacer, Mv Cam1 | Yes |
| pCFB115 | Type III Cas10HD mutant, gp43 spacer, gp Cam1 | Yes |
| pCFB116 | Type III Cas10HD mutant, gp43 spacer, Mv Cam1 D17E | Yes |
| pCFB117 | Type III Cas10HD mutant, gp43 spacer, Mv Cam1 D17K | Yes |
| pCFB118 | Type III Cas10HD mutant, gp43 spacer, Mv Cam1 D17S | Yes |
| pCFB119 | Type III Cas10HD mutant, gp43 spacer, Mv Cam1 D17A | Yes |
| pCFB121 | Type III Cas10HD mutant, gp43 spacer, gp Cam1 D22S | Yes |
| pCFB122 | Type III Cas10HD mutant, gp43 spacer, gp Cam1 D22E | Yes |
| pCFB123 | Type III Cas10HD mutant, gp43 spacer, gp Cam1 D22K | Yes |
| pCFB124 | Type III Cas10HD mutant, gp43 spacer, gp Cam1 D22A | Yes |
| pCFB125 | Type III Cas10HD mutant, gp43 spacer, Mv Cam1-Cterm-6xHis | Yes |
| pCFB126 | Type III Cas10HD mutant, gp43 spacer, gp Cam1-Cterm-6xHis | Yes |
| pCFB139 | Type III Cas10HD mutant, gp43 spacer, Nh Cam1 D17F | Yes |
| pCFB140 | Type III Cas10HD mutant, gp43 spacer, Nh Cam1 D17M | Yes |
| pCFB141 | Type III Cas10HD mutant, gp43 spacer, Nh Cam1 S24F | Yes |
| pCFB142 | Type III Cas10HD mutant, gp43 spacer, Mv Cam1 D17F | Yes |
| pCFB143 | Type III Cas10HD mutant, gp43 spacer, Mv Cam1 D17M | Yes |
| pCFB144 | Type III Cas10HD mutant, gp43 spacer, gp Cam1 D22F | Yes |
| pCFB145 | Type III Cas10HD mutant, gp43 spacer, gp Cam1 D22M | Yes |
| pJTR62 | Csm6-Cterm-6xHis in pE194 | No^1^ |
| pJTR109 | Type III Cas10HD mutant, gp43 spacer, Csm6 | No^1^ |
| pJTR162 | pTarget, aTc-inducible gp43 protospacer | No^1^ |
| pJTR396 | Type III Cas10HD mutant, gp43 spacer, Card1 | No^2^ |
| pJTR399 | Type III Cas10HD mutant, gp14 spacer, Nh Cam1 | Yes |
| pJTR404 | Type III, gp43 spacer, Nh Cam1 | Yes |
| pJTR407 | Type III Cas10HD mutant, ORF9 spacer, Nh Cam1 | Yes |
| pJTR408 | Type III, ORF9 spacer, Nh Cam1 | Yes |
| pJTR409 | Type III Cas10HD mutant, ORF27 spacer, Nh Cam1 | Yes |
| pJTR410 | Type III, ORF27 spacer, Nh Cam1 | Yes |
| pJTR447 | Type III, gp14 spacer, Nh Cam1 | Yes |
| pJTR448 | Type III Cas10HD mutant, gp43 spacer, Nh Cam1 | Yes |
| pJTR459 | Type III Cas10HD mutant, gp43 spacer, Nh Cam1 ΔTMH | Yes |
| pJTR460 | Type III Cas10HD mutant, gp43 spacer, Nh Cam1 ΔCARF | Yes |
| pJTR461 | Type III, BsaI spacer, Nh Cam1 | Yes |
| pJTR462 | Type III Cas10HD mutant, gp43 spacer, Nh Cam1-Cterm-6xHis | Yes |
|  |  |  |
