# Supplementary material for: The CRISPR effector Cam1 mediates membrane depolarization for phage defence
Source: Nature. 2024 Jan 10;625(7996):797–804. doi: 10.1038/s41586-023-06902-y (PMC10808066; doi:10.1038/s41586-023-06902-y)
Supplement: Supplementary file 8 — Oligonucleotide primers used in this study. [file 41586_2023_6902_MOESM8_ESM.docx]

**Supplementary Table 2.** Oligos used in this study

| **Name** | **Sequence** | **Purpose** |
| --- | --- | --- |
| CFB22 | ATCACCATCACCATCACTAAGACGTGGTTTAACCCGGGTAAC | Cloning |
| CFB21 | TTAGTGATGGTGATGGTGATGTCCTGAACCTTTTAAGTCCTCCTTAATAAAGAAAATAGG | Cloning |
| CFB23 | GCGACGGAGAGTTAGGTTATTGGG | Cloning |
| CFB24 | CCCAATAACCTAACTCTCCGTCGC | Cloning |
| GG424 | CATATTGCCTGATGAAGTGAATAG | Cloning |
| GG425 | CTATTCACTTCATCAGGCAATATG | Cloning |
| JTR715 | TTAAATTATACTATATTTTCCCATACCTAAGCTTG | Cloning |
| JTR716 | TTAAGACATAGTTAAAATTTAGTTGTCAAAAAATGTGAC | Cloning |
| W18 | TATTCTGAAAAGGTCAATCAAGG | Cloning |
| CFB62 | CCTTGATTGACCTTTTCAGAATAACTC | Cloning |
| CFB106 | CACTTTTAGTTTTAGCTAATATCATACTCAATCAGCCTCCCTTATTTGATAAATA | Cloning |
| CFB107 | TGAGTATGATATTAGCTAAAACTAAAAGTGG | Cloning |
| CFB108 | TTTAACCTCCATTTATATAAAGATTAGTTTTGGTTC | Cloning |
| CFB109 | AAACTAATCTTTATATAAATGGAGGTTAAAATGAATAAAAAAAATATATTAATGTATG | Cloning |
| W852 | CCAACAAACGACTTTTAGTATAACC | Cloning |
| W614 | GGTTATACTAAAAGTCGTTTGTTGG | Cloning |
| CFB118 | TTTACTGTCGCCGCCGAATCAGTACCGCGGTGGGTGATGGCACAAATTCAAC | Cloning |
| CFB119 | GTACTGATTCGGCGGCGACAGTAAATACAACACCCTCCCACTGCACTGTAGAC | Cloning |
| CFB124 | AATGAATCAGCCCCGCGGTGGGTGATGGCACAAATTCAACCCGC | Cloning |
| CFB125 | TCACCCACCGCGGGGCTGATTCATTACTGACAGTAAATACAACACCCTCCC | Cloning |
| CFB121 | GTTGCAGCCCAAGCTTCATTAGCAGCAGCAGAAGCTGTGGCTGCAGTGGCCCG | Cloning |
| CFB120 | GCTGCTGCTAATGAAGCTTGGGCTGCAACCAACCCCATATATGCGGGTTG | Cloning |
| CFB122 | CAGATGCTTATCAAAATCGGCGGCAACGGCCAGTGAAGCTACGCCGGCCTC | Cloning |
| CFB123 | TTGCCGCCGATTTTGATAAGCATCTGAAGGTGCCCGACATGCGGACTGCGACG | Cloning |
| CFB137 | GTATTTACTGTCAGTGCGGAATCAGTACCG | Cloning |
| CFB138 | CGGTACTGATTCCGCACTGACAGTAAATAC | Cloning |
| CFB135 | GTTGTATTTACTGTCGCGAATGAATCAGTACCG | Cloning |
| CFB136 | CGGTACTGATTCATTCGCGACAGTAAATACAAC | Cloning |
| CFB131 | ATCTGTGGCAACTGCCAGTGAAGCTAC | Cloning |
| CFB132 | CGGCGTAGCTTCACTGGCAGTTGCCACAGATTTTG | Cloning |
| CFB134 | CACTGTATGTTGCCGCGGATTTTGATAAGC | Cloning |
| CFB133 | TCAAAATCCGCGGCAACATACAGTGAA | Cloning |
| CFB224 | AAACTAATAAAAATAGCACTCCTAATCGTCATCTG | Cloning |
| CFB225 | AAAACAGATGACGATTAGGAGTGCTATTTTTATTA | Cloning |
| CFB257 | GTGCCCGACGCCCGGACTGCGACGTTACG | Cloning |
| CFB258 | ACGTCGCAGTCCGGGCGTCGGGCACCTTC | Cloning |
| CFB259 | TTGATAAGCATGCCAAGGTGCCCGACATGCGG | Cloning |
| CFB260 | GTCGGGCACCTTGGCATGCTTATCAAAATCTGTGGC | Cloning |
| CFB265 | GTGGATTTGACCGGTGGTGCCCTTCCAATGTCCCTG | Cloning |
| CFB266 | CAGGGACATTGGAAGGGCACCACCGGTCAAATCCAC | Cloning |
| CFB263 | GTATGTTGCCACAGATGCCGATAAGCATCTGAAGG | Cloning |
| CFB264 | CCTTCAGATGCTTATCGGCATCTGTGGCAACATAC | Cloning |
| CFB253 | GTGGCCGACGCCCGGACTGCGACGTTACGTCAG | Cloning |
| CFB254 | ACGTCGCAGTCCGGGCGTCGGCCACCTTCAGATGCTTATC | Cloning |
| CFB255 | GTGGCCGACATGCGGACTGCGACG | Cloning |
| CFB256 | CGTCGCAGTCCGCATGTCGGCCACCTTCAG | Cloning |
| CFB267 | TTGATAAGCATGCCAAGGTGCCCGACATGCGGACTGCGACG | Cloning |
| CFB268 | GCCACAGATTTTGATAAGCATGCCAAGGTGCCCGACATGCGGAC | Cloning |
| CFB402 | GGGCATCTTACTTGCAAACTTGGCATTCTTATTTG | Cloning |
| CFB403 | CAAATAAGAATGCCAAGTTTGCAAGTAAGATGCCCATTTGG | Cloning |
| CFB412 | GAAAGTAAGATGCCCATTTGTGATAACAGCAGCCAGATTCCG | Cloning |
| CFB413 | CGGAATCTGGCTGCTGTTATCACAAATGGGCATCTTACTTTCAAAC | Cloning |
| CFB400 | GTAAGATGCCCATTTGTGCTAACAGCAGCCAGATTC | Cloning |
| CFB401 | CTGGCTGCTGTTAGCACAAATGGGCATCTTACTTTC | Cloning |
| CFB407 | CGCAAATAAGAATGCCAAGTTTTTAAGTAAGATGCCCATTTGGTC | Cloning |
| CFB406 | CAAATGGGCATCTTACTTAAAAACTTGGCATTCTTATTTGCGC | Cloning |
| CFB419 | AAAGTAAGATGCCCATTTGTTCTAACAGCAGCCAGATTC | Cloning |
| CFB418 | GAATCTGGCTGCTGTTAGAACAAATGGGCATCTTACTTTC | Cloning |
| CFB423 | GTTTGAAAGTAAGATGCCCATTTGCCGTAACAGCAGCCAGATTCCG | Cloning |
| CFB422 | GGAATCTGGCTGCTGTTACGGCAAATGGGCATCTTACTTTCAAAC | Cloning |
| CFB425 | GCAAATAAGAATGCCAAGTTCCGAAGTAAGATGCCCATTTGGTC | Cloning |
| CFB424 | GACCAAATGGGCATCTTACTTCGGAACTTGGCATTCTTATTTGC | Cloning |
| CFB421 | AAAGTAAGATGCCCATTTGGTTTAACAGCAGCCAGATTC | Cloning |
| CFB420 | GAATCTGGCTGCTGTTAAACCAAATGGGCATCTTACTTTC | Cloning |
| CFB427 | CGCAAATAAGAATGCCAAGTTCATAAGTAAGATGCCCATTTGGTCTAAC | Cloning |
| CFB426 | GACCAAATGGGCATCTTACTTAGTAACTTGGCATTCTTATTTGCG | Cloning |
| CFB466 | GCAAATAAGAATGCCAAGTTGTCAAGTAAGATGCCCATTTG | Cloning |
| CFB467 | CAAATGGGCATCTTACTTGACAACTTGGCATTCTTATTTGC | Cloning |
| CFB415 | GCAAATAAGAATGCCAAGTTGTCAAGTAAGATGCCCATTTGGTCTAACAG | Cloning |
| CFB414 | GACCAAATGGGCATCTTACTTGACAACTTGGCATTCTTATTTGCGC | Cloning |
| CFB151 | GGTATGGGAAAATATAGTATAATTTAAATTTAAGAAGGAGATATACATATGCGTGCG | Cloning |
| CFB432 | TAATTAAGACATAGTTAAAATTTAGTTGTCAAAAAATGTGAC | Cloning |
| CFB472 | GTATAATTTAAATTTAAGAAGGAGATATACATATGAACGAACAATTATTTGGTTGGAACG | Cloning |
| CFB473 | TTGACAACTAAATTTTAACTATGTCTTAATTATTGTGTACGACTAACTGACGTGATACTC | Cloning |
| CFB474 | TATAGTATAATTTAAATTTAAGAAGGAGATATACATATGTATTTTACAGGCGCAAACGGC | Cloning |
| CFB475 | ATTTTTTGACAACTAAATTTTAACTATGTCTTAATTAACTTTTCGCGTGCGTGATACAAC | Cloning |
| CFB487 | CCTGCTAAGATGCCTAACTGTTCTAAATACAACCATGTCTGC | Cloning |
| CFB486 | GCAGACATGGTTGTATTTAGAACAGTTAGGCATCTTAGCAGG | Cloning |
| CFB489 | CCTGCTAAGATGCCTAACTGTTTTAAATACAACCATGTCTGC | Cloning |
| CFB488 | GCAGACATGGTTGTATTTAAAACAGTTAGGCATCTTAGCAGG | Cloning |
| CFB485 | CCTGCTAAGATGCCTAACTGTGATAAATACAACCATGTCTGC | Cloning |
| CFB484 | GCAGACATGGTTGTATTTATCACAGTTAGGCATCTTAGCAGG | Cloning |
| CFB491 | CCTGCTAAGATGCCTAACTGTGCTAAATACAACCATGTCTGC | Cloning |
| CFB490 | GCAGACATGGTTGTATTTAGCACAGTTAGGCATCTTAGCAGG | Cloning |
| CFB497 | CTAACAAAATACCGAACTGAGATAACCACAACCACGTAG | Cloning |
| CFB496 | CTACGTGGTTGTGGTTATCTCAGTTCGGTATTTTGTTAG | Cloning |
| CFB499 | CTAACAAAATACCGAACTGCTCTAACCACAACCACGTAGAC | Cloning |
| CFB498 | GTCTACGTGGTTGTGGTTAGAGCAGTTCGGTATTTTGTTAG | Cloning |
| CFB501 | CTAACAAAATACCGAACTGTTTTAACCACAACCACGTAGAC | Cloning |
| CFB500 | GTCTACGTGGTTGTGGTTAAAACAGTTCGGTATTTTGTTAG | Cloning |
| CFB503 | CTAACAAAATACCGAACTGCGCTAACCACAACCACGTAGAC | Cloning |
| CFB502 | GTCTACGTGGTTGTGGTTAGCGCAGTTCGGTATTTTGTTAG | Cloning |
| CFB493 | GTGATGGTGATGGTGATGTCCTGAACCTTGTGTACGACTAACTGACGTGATACTCG | Cloning |
| CFB458 | GGTCATCAATTTTTGTCCCAATTTTCAGAATTTAAAGACAAC | Cloning |
| CFB459 | GTTGTCTTTAAATTCTGAAAATTGGGACAAAAATTGATGACC | Cloning |
| CFB494 | GGTTCAGGACATCACCATCACCATCACTAATTAAGACATAGTTAAAATTTAGTTGTCAAAAAATGTGAC | Cloning |
| CFB597 | GAAAGTAAGATGCCCATTTGAAATAACAGCAGCCAGATTCC | Cloning |
| CFB596 | GGAATCTGGCTGCTGTTATTTCAAATGGGCATCTTACTTTC | Cloning |
| CFB599 | GAAAGTAAGATGCCCATTTGCATTAACAGCAGCCAGATTCC | Cloning |
| CFB598 | GGAATCTGGCTGCTGTTAATGCAAATGGGCATCTTACTTTC | Cloning |
| CFB601 | GCAAATAAGAATGCCAAGTTAAAAAGTAAGATGCCCATTTG | Cloning |
| CFB600 | CAAATGGGCATCTTACTTTTTAACTTGGCATTCTTATTTGC | Cloning |
| CFB589 | CCTGCTAAGATGCCTAACTGGAATAAATACAACCATGTCTGC | Cloning |
| CFB588 | GCAGACATGGTTGTATTTATTCCAGTTAGGCATCTTAGCAGG | Cloning |
| CFB591 | CCTGCTAAGATGCCTAACTGCATTAAATACAACCATGTCTGC | Cloning |
| CFB590 | GCAGACATGGTTGTATTTAATGCAGTTAGGCATCTTAGCAGG | Cloning |
| CFB593 | CTAACAAAATACCGAACTGGAATAACCACAACCACGTAGAC | Cloning |
| CFB592 | GTCTACGTGGTTGTGGTTATTCCAGTTCGGTATTTTGTTAG | Cloning |
| CFB595 | CTAACAAAATACCGAACTGCATTAACCACAACCACGTAGAC | Cloning |
| CFB594 | GTCTACGTGGTTGTGGTTAATGCAGTTCGGTATTTTGTTAG | Cloning |
| JTR952 | GAAAGTTTAAATACTTTTGTTGCCAATGTCATTCACCTACTTAATTTTAAGATTTG | Cloning |
| JTR951 | CAACAAAAGTATTTAAACTTTCTTTTAAGACTC | Cloning |
| JTR775 | AAGGAATTTAAAGAGCTATTATGATAAATAAAATTACAG | Cloning |
| JTR776 | ATCATAATAGCTCTTTAAATTCCTTATTATAGCACCTCATTATTTAACTCTTGAAAAC | Cloning |
| JTR401 | CGACTATGCTAGTCAAAATCAAGATATGAG | Cloning |
| JTR402 | CTCATATCTTGATTTTGACTAGCATAGTCG | Cloning |
| JTR872 | CATATGTATATCTCCTTCTTAAATTTAAATTATACTATATTTTCCC | Cloning |
| JTR873 | TTTAAATTTAAGAAGGAGATATACATATGCGGCGCGAAGATCTTCGGG | Cloning |
| JTR874 | CTAAATTTTAACTATGTCTTAATTAGGTTAACCATGCCCGAAG | Cloning |
| JTR868 | TAATTAAGACATAGTTAAAATTTAGTTGTCAAAAAATGTG | Cloning |
| JTR1027 | CTAAATTTTAACTATGTCTTAATTAGTGATGGTGATGGTGATGTCCTGAACCCTCCGGTTGGCTAATCTGACG | Cloning |
| JTR716 | TTAAGACATAGTTAAAATTTAGTTGTCAAAAAATGTGAC | Cloning |
