# Supplementary material for: The CRISPR effector Cam1 mediates membrane depolarization for phage defence
Source: Nature. 2024 Jan 10;625(7996):797–804. doi: 10.1038/s41586-023-06902-y (PMC10808066; doi:10.1038/s41586-023-06902-y)
Supplement: Supplementary file 9 — Cloning strategies used in this study. [file 41586_2023_6902_MOESM9_ESM.docx]

**Supplementary Table 3.** Cloning strategies used in this study

| **Name** | **Cloning strategy** |
| --- | --- |
| pCFB1 | PCR amplification of pJTR411 with CFB21 and CFB23, and of pJTR411 with CFB22 and CFB24, followed by Gibson assembly of the two PCR products |
| pCFB2 | PCR amplification of pJTR404 with JTR716 and GG425, and of pJTR404 with JTR715 and GG424, followed by Gibson assembly of the two PCR products |
| pCFB5 | PCR amplification of pJTR410 with JTR716 and GG425, and of pJTR410 with JTR715 and GG424, followed by Gibson assembly of the two PCR products |
| pCFB6 | PCR amplification of pCFB4 with W18 and GG425, and of pCFB4 with CFB62 and GG424, followed by Gibson assembly of the two PCR products |
| pCFB20 | PCR amplification of pJTR394 with CFB109 and CFB106, and of pJTR448 with CFB108 and CFB107, followed by Gibson assembly of the two PCR products |
| pCFB25 | PCR amplification of pCFB22 with W852 and GG425, and of pCFB2 with W614 and GG424, followed by Gibson assembly of the two PCR products |
| pCFB26 | PCR amplification of pJTR448 with CFB118 and GG425, and of pJTR448 with CFB119 and GG424, followed by Gibson assembly of the two PCR products |
| pCFB27 | PCR amplification of pJTR448 with CFB124 and GG425, and of pJTR448 with CFB125 and GG424, followed by Gibson assembly of the two PCR products |
| pCFB28 | PCR amplification of pJTR448 with CFB121 and GG425, and of pJTR448 with CFB120 and GG424, followed by Gibson assembly of the two PCR products |
| pCFB29 | PCR amplification of pJTR448 with CFB123 and GG425, and of pJTR448 with CFB122 and GG424, followed by Gibson assembly of the two PCR products |
| pCFB31 | PCR amplification of pJTR448 with CFB137 and GG425, and of pJTR448 with CFB138 and GG424, followed by Gibson assembly of the two PCR products |
| pCFB32 | PCR amplification of pJTR448 with CFB135 and GG425, and of pJTR448 with CFB136 and GG424, followed by Gibson assembly of the two PCR products |
| pCFB33 | PCR amplification of pJTR448 with CFB132 and GG425, and of pJTR448 with CFB131 and GG424, followed by Gibson assembly of the two PCR products |
| pCFB34 | PCR amplification of pJTR448 with CFB134 and GG425, and of pJTR448 with CFB133 and GG424, followed by Gibson assembly of the two PCR products |
| pCFB55 | PCR amplification of pAS23 with CFB213 and PM783, φNM1 genomic DNA with CFB214 and CFB215, pAS23 with CFB216 and CFB217, and φNM1 with CFB218 and CFB219, followed by Gibson assembly of the four PCR products |
| pCFB58 | BsaI-HF (NEB) digestion of plasmid pDB114, followed by ligation with annealed oligos CFB224 and CFB225 with compatible overhangs |
| pCFB64 | PCR amplification of pJTR448 with CFB257 and GG425, and of pJTR448 with CFB258 and GG424, followed by Gibson assembly of the two PCR products |
| pCFB65 | PCR amplification of pJTR448 with CFB259 and GG425, and of pJTR448 with CFB260 and GG424, followed by Gibson assembly of the two PCR products |
| pCFB67 | PCR amplification of pJTR448 with CFB265 and GG425, and of pJTR448 with CFB266 and GG424, followed by Gibson assembly of the two PCR products |
| pCFB68 | PCR amplification of pJTR448 with CFB263 and GG425, and of pJTR448 with CFB264 and GG424, followed by Gibson assembly of the two PCR products |
| pCFB71 | PCR amplification of pCFB27 with CFB253 and JTR402, and of pCFB27 with CFB254 and JTR401, followed by Gibson assembly of the two PCR products |
| pCFB72 | PCR amplification of pJTR448 with CFB255 and JTR402, and of pCFB448 with CFB256 and JTR401, followed by Gibson assembly of the two PCR products |
| pCFB73 | PCR amplification of pCFB67 with CFB267 and JTR402, and of pCFB67 with CFB268 and JTR401, followed by Gibson assembly of the two PCR products |
| pCFB92 | PCR amplification of pJTR448 with CFB402 and GG425, and of pJTR448 with CFB403 and GG424, followed by Gibson assembly of the two PCR products |
| pCFB93 | PCR amplification of pJTR448 with CFB412 and GG424, and of pJTR448 with CFB413 and GG425, followed by Gibson assembly of the two PCR products |
| pCFB95 | PCR amplification of pJTR448 with CFB400 and GG424, and of pJTR448 with CFB401 and GG425, followed by Gibson assembly of the two PCR products |
| pCFB96 | PCR amplification of pJTR448 with CFB407 and GG424, and of pJTR448 with CFB406 and GG425, followed by Gibson assembly of the two PCR products |
| pCFB98 | PCR amplification of pJTR448 with CFB419 and GG424, and of pJTR448 with CFB418 and GG425, followed by Gibson assembly of the two PCR products |
| pCFB99 | PCR amplification of pJTR448 with CFB423 and GG424, and of pJTR448 with CFB422 and GG425, followed by Gibson assembly of the two PCR products |
| pCFB100 | PCR amplification of pJTR448 with CFB425 and GG424, and of pJTR448 with CFB424 and GG425, followed by Gibson assembly of the two PCR products |
| pCFB101 | PCR amplification of pJTR448 with CFB421 and GG424, and of pJTR448 with CFB420 and GG425, followed by Gibson assembly of the two PCR products |
| pCFB102 | PCR amplification of pJTR448 with CFB427 and GG424, and of pJTR448 with CFB426 and GG425, followed by Gibson assembly of the two PCR products |
| pCFB103 | PCR amplification of pJTR448 with CFB466 and GG424, and of pJTR448 with CFB467 and GG425, followed by Gibson assembly of the two PCR products |
| pCFB104 | PCR amplification of pJTR448 with CFB415 and GG424, and of pJTR448 with CFB414 and GG425, followed by Gibson assembly of the two PCR products |
| pCFB114 | PCR amplification of pJTR448 with CFB151 and CFB432, and of a gblock of M. vadi Cam1 with CFB472 and CFB473, followed by Gibson assembly of the two PCR products |
| pCFB115 | PCR amplification of pJTR448 with CFB151 and CFB432, and of a gblock of unknown gammaproteobacteria Cam1 with CFB474 and CFB475, followed by Gibson assembly of the two PCR products |
| pCFB116 | PCR amplification of pCFB114 with CFB487 and GG424, and of pCFB114 with CFB486 and GG425, followed by Gibson assembly of the two PCR products |
| pCFB117 | PCR amplification of pCFB114 with CFB489 and GG424, and of pCFB114 with CFB488 and GG425, followed by Gibson assembly of the two PCR products |
| pCFB118 | PCR amplification of pCFB114 with CFB485 and GG424, and of pCFB114 with CFB484 and GG425, followed by Gibson assembly of the two PCR products |
| pCFB119 | PCR amplification of pCFB114 with CFB491 and GG424, and of pCFB114 with CFB490 and GG425, followed by Gibson assembly of the two PCR products |
| pCFB121 | PCR amplification of pCFB115 with CFB497 and GG424, and of pCFB115 with CFB496 and GG425, followed by Gibson assembly of the two PCR products |
| pCFB122 | PCR amplification of pCFB115 with CFB499 and GG424, and of pCFB115 with CFB498 and GG425, followed by Gibson assembly of the two PCR products |
| pCFB123 | PCR amplification of pCFB115 with CFB501 and GG424, and of pCFB115 with CFB500 and GG425, followed by Gibson assembly of the two PCR products |
| pCFB124 | PCR amplification of pCFB115 with CFB503 and GG424, and of pCFB115 with CFB502 and GG425, followed by Gibson assembly of the two PCR products |
| pCFB125 | PCR amplification of pCFB114 with CFB493 and CFB458, and of pCFB114 with CFB459 and CFB492, followed by Gibson assembly of the two PCR products |
| pCFB126 | PCR amplification of pCFB115 with CFB495 and CFB458, and of pCFB115 with CFB459 and CFB494, followed by Gibson assembly of the two PCR products |
| pCFB139 | PCR amplification of pJTR448 with CFB597 and GG424, and of pJTR448 with CFB596 and GG425, followed by Gibson assembly of the two PCR products |
| pCFB140 | PCR amplification of pJTR448 with CFB599 and GG424, and of pJTR448 with CFB598 and GG425, followed by Gibson assembly of the two PCR products |
| pCFB141 | PCR amplification of pJTR448 with CFB601 and GG424, and of pJTR448 with CFB600 and GG425, followed by Gibson assembly of the two PCR products |
| pCFB142 | PCR amplification of pCFB114 with CFB589 and GG424, and of pCFB114 with CFB588 and GG425, followed by Gibson assembly of the two PCR products |
| pCFB143 | PCR amplification of pCFB114 with CFB591 and GG424, and of pCFB114 with CFB590 and GG425, followed by Gibson assembly of the two PCR products |
| pCFB144 | PCR amplification of pCFB115 with CFB593 and GG424, and of pCFB115 with CFB592 and GG425, followed by Gibson assembly of the two PCR products |
| pCFB145 | PCR amplification of pCFB115 with CFB595 and GG424, and of pCFB115 with CFB594 and GG425, followed by Gibson assembly of the two PCR products |
| pJTR399 | PCR amplification of pJTR119 with W852 and JTR952, and of pJTR385 with JTR951 and W614, followed by Gibson assembly of the two PCR products |
| pJTR404 | PCR amplification of pWJ241 with JTR775 and W614, and of pJTR385 with JTR776 and W852, followed by Gibson assembly of the two PCR products |
| pJTR407 | PCR amplification of pJTR169 with W852 and GG425, and of pJTR385 with W614 and GG424, followed by Gibson assembly of the two PCR products |
| pJTR408 | PCR amplification of pJTR169 with W852 and GG425, and of pJTR404 with W614 and GG424, followed by Gibson assembly of the two PCR products |
| pJTR409 | PCR amplification of pJTR170 with W852 and GG425, and of pJTR385 with W614 and GG424, followed by Gibson assembly of the two PCR products |
| pJTR410 | PCR amplification of pJTR170 with W852 and GG425, and of pJTR404 with W614 and GG424, followed by Gibson assembly of the two PCR products |
| pJTR447 | PCR amplification of pWJ245 with W852 and JTR402, and of pJTR399 with W614 and JTR401, followed by Gibson assembly of the two PCR products |
| pJTR448 | PCR amplification of pJTR109 with W852 and JTR402, and of pJTR399 with W614 and JTR401, followed by Gibson assembly of the two PCR products |
| pJTR459 | PCR amplification of pJTR448 with W852 and JTR872, and of pJTR448 with W614 and JTR873, followed by Gibson assembly of the two PCR products |
| pJTR460 | PCR amplification of pJTR448 with W852 and JTR874, and of pJTR448 with W614 and JTR868, followed by Gibson assembly of the two PCR products |
| pJTR461 | PCR amplification of pGG-BsaI-R with W852 and JTR402, and of pJTR399 with W614 and JTR401, followed by Gibson assembly of the two PCR products |
| pJTR462 | PCR amplification of pJTR448 with W852 and JTR1027, and of pJTR448 with W614 and JTR716, followed by Gibson assembly of the two PCR products |
