# Supplementary material for: The CRISPR effector Cam1 mediates membrane depolarization for phage defence
Source: Nature. 2024 Jan 10;625(7996):797–804. doi: 10.1038/s41586-023-06902-y (PMC10808066; doi:10.1038/s41586-023-06902-y)
Supplement: Supplementary file 10 — Structural data collection and refinement statistics. [file 41586_2023_6902_MOESM10_ESM.docx]

**Supplementary Table 4.** Data collection and refinement statistics.

|  | Apo Cam1  (PDB 8T64) | cA4-Cam1  (PDB 8T65) | cA6-Cam1  (PDB 8T66) |
| --- | --- | --- | --- |
| Data collection | | | |
| Wavelength (Å) | 0.9792 | 0.9792 | 0.9792 |
| Space group | P1211 | P1211 | C222_1_ |
| Cell dimensions | | | |
| a, b, c (Å) | 43.8, 120.6, 51.5 | 47.0, 64.9, 50.1 | 70.4, 75.8, 125.2 |
| α, β, γ (°) | 90, 115.3, 90 | 90, 113.3, 90 | 90, 90, 90 |
| Resolution (Å) | 50.00-2.25  (2.33-2.25) | 64.86-2.09  (2.15-2.09) | 125.20-1.85  (1.89 -1.85) |
| R-merge | 0.083 (0.267) | 0.057 (0.249) | 0.209 (0.948) |
| R-pim | 0.039(0.125) | 0.037 (0.166) | 0.075(0.392) |
| I/σI | 24.3 (4.9) | 14.6 (4.3) | 19.4 (2.2) |
| Completeness (%) | 97.8 (95.0) | 97.3 (72.6) | 99.1 (86.2) |
| Redundancy | 4.3 (4.2) | 4.4 (3.9) | 8.7 (7.3) |
| CC1/2 | 0.996 (0.955) | 0.998 (0.938) | 0.998 (0.784) |
| Unique  Reflections | 22,339 (2,199) | 16,018 (1387) | 28,642 (2,589) |
| Refinement | | | |
| Rwork/Rfree (%) | 20.6/24.5 | 17.3/18.3 | 19.6/22.2 |
| Reflections in refinement | 22,326 (2,198) | 15,995, (1,383) | 28,606 (2,561) |
| No. of non-hydrogen atoms | | | |
| Proteins | 3,904 | 2,300 | 2,316 |
| cAn | -- | 88 | 110 |
| Water | 98 | 128 | 166 |
| Protein residues | 534 | 308 | 311 |
| B-factors (Å2) | | | |
| Proteins | 46.0 | 32.5 | 26.7 |
| cAn | -- | 32.7 | 41.1 |
| Water | 44.4 | 40.2 | 33.6 |
| R.m.s. deviations | | | |
| Bond lengths (Å) | 0.013 | 0.009 | 0.008 |

| Bond angles (°) | 1.18 | 0.92 | 0.97 |
| --- | --- | --- | --- |
| Ramachandran plots | | | |
| Favored (%) | 98.46 | 99.01 | 99.02 |
| Allowed (%) | 1.54 | 0.99 | 0.98 |
| Outliers (%) | 0.00 | 0.00 | 0.00 |
| Rotamer outliers  (%) | 2.81 | 0.00 | 0.42 |
| Clashscore | 9.83 | 3.58 | 3.54 |
| Statistics for the highest-resolution shell are shown in parenthesis. | | | |
